# Integrating Time-Resolved *nrf2* Gene-Expression Data into a Full GUTS Model as a Proxy for Toxicodynamic Damage in Zebrafish Embryo

**DOI:** 10.1021/acs.est.4c06267

**Published:** 2024-12-04

**Authors:** Florian Schunck, Bernhard Kodritsch, Martin Krauss, Wibke Busch, Andreas Focks

**Affiliations:** † Osnabrück University, Barbarastr. 12, 49076 Osnabrück, Germany; ‡ Helmholtz-Centre for Environmental Research GmbH−UFZ, Permoserstr. 15, 04318 Leipzig, Germany

**Keywords:** modeling, molecular TKTD, RNA, transcriptomics, Bayesian, toxicogenomics, stress-response

## Abstract

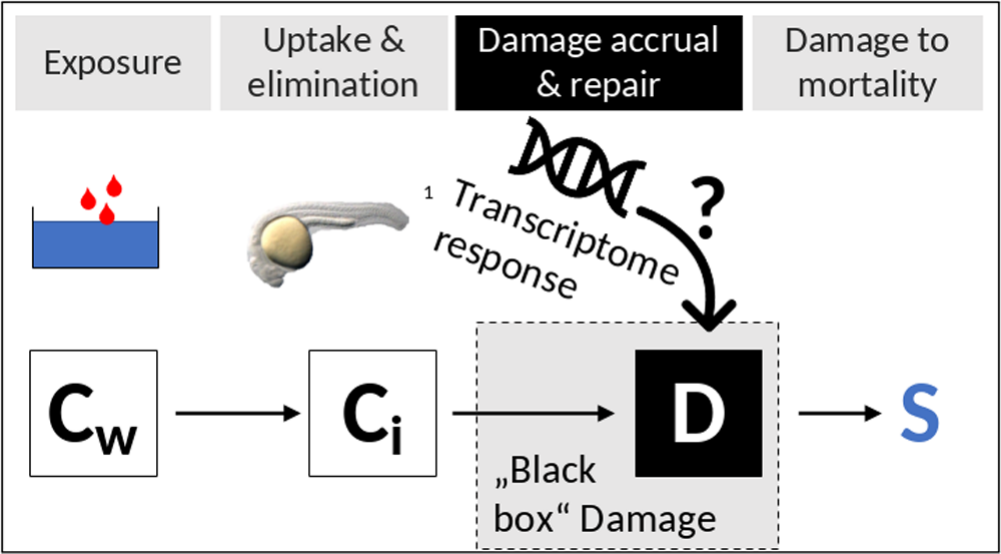

The immense production of the chemical industry requires an improved predictive risk assessment that can handle constantly evolving challenges while reducing the dependency of risk assessment on animal testing. Integrating omics data into mechanistic models offers a promising solution by linking cellular processes triggered after chemical exposure with observed effects in the organism. With the emerging availability of time-resolved RNA data, the goal of integrating gene expression data into mechanistic models can be approached. We propose a biologically anchored TKTD model, which describes key processes that link the gene expression level of the stress regulator *nrf2* to detoxification and lethality by associating toxicodynamic damage with *nrf2* expression. Fitting such a model to complex data sets consisting of multiple endpoints required the combination of methods from molecular biology, mechanistic dynamic systems modeling, and Bayesian inference. In this study, we successfully integrate time-resolved gene expression data into TKTD models and thus provide a method for assessing the influence of molecular markers on survival. This novel method was used to test whether *nrf2* can be applied to predict lethality in zebrafish embryos. With the presented approach, we outline a method to successfully approach the goal of a predictive risk assessment based on molecular data.

## Abstract 2

Integrating dynamic omics data into TKTD models advances understanding of effect pathways and progress toward predicting untested chemicals

## Introduction

The immense production of the chemical industry^1^ and the resulting release of novel substances into the environment^2^ require an improved predictive risk assessment that can handle constantly renewing challenges. Tackling this problem experimentally has blind spots with respect to potentially vulnerable species (e.g., pollinator decline,^3−5^ sublethal effects of chemicals, and mixtures). The sheer combinatorial complexity of the problem precludes testing as a strategy. In silico approaches can be one way forward to achieve a prospective risk assessment and at the same time reduce an immense need for animal testing if all of the above issues should be addressed. While data-driven approaches like QSARs and deep learning display their power, they are constrained by the data they are calibrated to, hence extrapolation is limited.^6,7^ Mechanistic models encode causal relationships through processes over time, and are in this way capable of answering higher-order questions such as “What if?”, “Why?”, or “How would it look like under changed conditions?”.^7^ Such models can be designed when biophysical processes are deciphered (advances in molecular biology), causal relationships are defined through temporal sequences (mathematical abstraction), and data become available to learn the dominant processes that drive toxicity in humans or environmental organisms.

The growing availability of omics data drives the abstraction of biophysical insights into the processes that govern molecular responses to changing environments.^8^ The integration of omics data into mechanistic models therefore offers a promising solution for advancing risk assessment for chemicals and chemical mixtures because, in theory, it can connect the cellular processes induced after toxicant exposure with observed effects in the organism.^9,10^ Developing such approaches envisions the prediction of toxicant effects for untested species to substance combinations and mixtures as a very desirable long-term goal for a predictive environmental risk assessment.

It was recently shown that gene expression data from single time-point measurements^11^ can be integrated into mechanistic models.^12^ The next critical step is the integration of temporally resolved omics data to also describe the dynamics of intermediate processes. To simultaneously model the dynamics of multiple process steps, models have to account for the relevant biological processes, aiming to enhance model accuracy and understand the intermediate processes that lead to the observed effects. Compared to the efforts and costs of animal studies, which rarely provide time-resolved data, omics-assisted mechanistic models can offer a valuable and intriguing perspective for in vitro bioassays.

To advance prospective risk assessment, the challenge arises to develop general models that are firmly grounded in biology and can integrate the temporal dynamics of multiple stages in the toxicant response, including molecular responses.

Toxicokinetic-toxicodynamic (TKTD) models seem ideally suited to facilitate the proposed integration of omics data into mechanistic models. They consider the uptake and elimination kinetics of chemicals (toxicokinetics, TK) and translate internal concentrations or other dose metrics to dynamic toxic effects (toxicodynamics, TD). TKTD models are frequently applied to model toxic effects over time,^13^ and interactions between chemicals.^14−17^ Particularly the general unified threshold model for survival (GUTS)^18^ is a commonly used TKTD framework to model time-resolved survival data and even effects of chemical mixtures over time.^19^ Most importantly, GUTS models include a damage state, which responds to internal toxicant concentrations and represents an impact state inside an organism, from which observable effects follow. This damage state is abstract, but as well might also be the state variable that corresponds most with omics data. Further investigation of the potential correspondence between the GUTS damage state and omics data is very challenging because of the limited availability of data sets that include both toxicokinetic, molecular, and apical endpoints over time. In this study, we address this challenge and suggest a model structure that integrates gene expression data into TKTD models to approximate the damage state.

A central pathway involved in the translation of environmental concentrations to observable effects is the integrated stress response (ISR).^20^ It is an intracellular signaling network that helps cells and organisms to maintain health in a variable environment. It modulates cellular processes, among them mRNA translation and metabolism to enable cells to repair damage,^21^ or if damage repair is unsuccessful, triggers apoptosis to remove damaged cells. In the cellular stress response to chemical exposure, Nrf2 has been identified as a master regulator of the detoxification process and its signaling pathway has been extensively described.^22,23^ Under basal conditions, *nrf2* transcription and synthesis rates to Nrf2 proteins are kept in balance by KEAP1 proteins and ubiquitination targeted degradation^24,25^ with a half-life of approximately 10–20 min.^24,26^ Nrf2 activation is tightly linked to the AhR pathway, which is also known to be one of the major chemical-induced metabolic pathways, and to the KEAP1 pathway, known to play a key role in oxidative stress response in organisms.^27^ Upon activation, Nrf2 translocates into the nucleus and activates the transcription of genes that remediate stress via interaction with antioxidant response elements (ARE)s. Nrf2 activation can, therefore, be understood as a proxy indicative of stress induced by chemical exposure and related toxicity. In a recently published study, the transcriptome of zebrafish embryos (ZFE) was measured at multiple points in time, after exposure to toxicants.^28^ The regulation frequency of various gene clusters was temporally related to the internal concentration profiles in ZFE, indicating the value of gene-expression data in modeling the response to toxicants. Early work showed promising results in this area.^29^ Typical pulse-like expression profiles^30,31^ were observed in a gene cluster containing the *nfe2L2b* gene (from here referred to as *nrf2*), which expresses the Nrf2 protein in zebrafish, indicating that active reduction of damage needs to be considered in the modeling when integrating molecular responses such as *nrf2* expression into TKTD models.

We hypothesize that toxicodynamic damage can be approximated by gene-expression data and thus serve as an interface for integrating omics data into mechanistic models. To investigate this hypothesis, we use the time-resolved *nrf2* expression signal from the published data set of^28^ complemented with unpublished time-series data of internal toxicant concentrations and survival and assess the potential of integrating it into TKTD models.

We further hypothesize that the expression of stress regulator gene *nrf2* can be used to model lethality in zebrafish embryos independent of specific toxicant characteristics such as the mode of action. For this, we implement a parameter-sharing^15^ approach, where a combined model is fitted to data from multiple substances with substance-specific parameters for uptake, and general parameters for the gene expression and protein dynamics. Typically, TKTD models are parametrized on single-substance single-species data sets, but we propose that by integrating omics data as a damage proxy, this paradigm can be overcome for a subset of the parameters. To test this hypothesis, we compare the Bayesian information criterion (BIC) of the combined (substance-independent) model to a model in which all parameters are fitted independently (substance-specific).

This study provides a biologically anchored TKTD model, which links the gene expression level of *nrf2* to detoxification and lethality and in that way replaces the damage state in the standard GUTS models.

## Methods

### Description of the GUTS-RNA-Pulse Model

**Figure 1:**
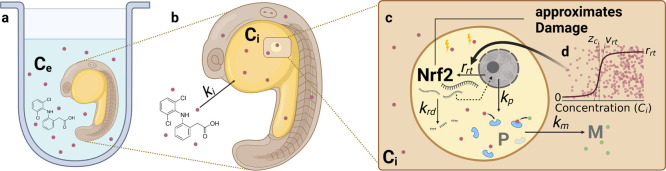
Graphical description of the GUTS-RNA-pulse model, where the *nrf2* concentration in the whole organism is used as a proxy for toxicodynamic damage. (a) Zebrafish embryo exposed to a chemical from 24–120 h post fertilization (hpf). (b) Uptake of diclofenac (exemplarily) into the organism at rate constant *k*_i_ (eq 4). (c) Zoom into the hypothesized expression metabolization process: *nrf2* (*R*) is expressed at a constant rate *r*_rt_ with a responsiveness *v*_rt_ when the internal substance concentration *C*_i_ exceeds a substance specific threshold *z*_ci_ (shown in d) and decays at a concentration dependent rate constant *k*_rd_ (eq 1). Unobserved metabolizing protein dynamics depend on the *nrf2* concentration and are described with a dominant rate constant *k*_p_ (eq 3). Metabolization of the chemical depends on the protein concentrations *P*, *C*_i_, and the metabolization rate constant *k*_m_ (eq 4). The nonmonotonically increasing level of *nrf2* is used to approximate toxicodynamic damage and is linked to ZFE survival via the stochastic death model by eqs 5 and 6. Created with BioRender.com.

In this study, several toxicokinetic-toxicodynamic (TKTD) models are compared. Here, we focus on describing the integration of RNA expression into a GUTS TKTD model (named the GUTS-RNA-pulse hereafter). In the model, a constant *nrf2* expression rate follows a concentration-dependent activation (Figure 1). *Nrf2* is assumed to be indirectly responsible for the metabolization of the chemical in the organism and is also linked to survival via a threshold model. A comprehensive list of all state variables and parameters is given in Table 1.

**Table 1:** TKTD State Variables and Parameters Used in the GUTS-RNA-Pulse Model^a^

symbol	definition	unit	substance independent
Model state variables			
*C*_e_	environmental concentration in the aqueous medium	μmol L^–1^	
*C*_i_	internal concentration of the homogenized ZFE	μmol L^–1^	
*R*	relative differential RNA transcription in the ZFE	fc^d^	
*R*_0_	relative differential initial RNA transcription in the ZFE	fc^d^	
P*	scaled protein concentration in the ZFE	fc^d^	
*h*	instantaneous hazard rate at time *t*	h^–1^	
*S*	survival probability of a ZFE at time *t*		
Model parameters			
*k*_i_	uptake rate constant of the chemical into the internal compartment	h^–1^	no
*k*_m_	scaled metabolization rate constant from the internal compartment	h^–1^	no
*z*_ci_	scaled *C*_i_ threshold for the activation of *nrf2* expression	e	no
*v*_rt_	scaled responsiveness of the *nrf2* activation (slope of the activation)	e	yes/no^b^
*r*_rt_	constant *nrf2* expression rate after activation^c^	fc^d^	yes
*k*_rd_	*nrf2* decay rate constant	h^–1^	yes
*k*_p_	degradation rate constant of metabolizing proteins	h^–1^	yes
*z*	effect *nrf2*-threshold of the hazard function^c^	fc^d^	yes
*k*_k_	killing rate constant for *nrf2*^c^	fc^–1^ h^–1^^d^	yes
*h*_b_	background hazard rate	h^–1^	yes
σ_cint_	std. dev. of the log-normal distribution of the internal concentration	f	yes
σ_*nrf*2_	std. dev. of the log-normal distribution of the *nrf2* expression^c^	f	yes

a The column “Substance independent” indicates whether a parameter is supposed to be shared for multiple substances.

b In an unscaled version of the activation function, *v*_rt_ is not considered substance independent, due to an inverse relationship between *v*_rt_ and *C*_i,max_.

c Relative to the *nrf2* concentration in untreated ZFE (fold-change).

d fc: fold change .

e Scaled internal concentrations: .

f Follows the random variable’s dimension on the linear scale but is dimensionless on the log scale.

#### *nrf2* Activation

The chemical stress response with respect to *nrf2* regulation is complex. There are several *nrf2* regulating signaling pathways, including Nrf2 dissociation from KEAP1 and stabilization of Nrf2 proteins in the cytosol, Nrf2 autoregulation, and AhR-induced response.^27^ To generalize, the model focuses on commonly observed gene-expression patterns, which are (1) short expression impulses and (2) sustained expression.^30,31^ Pulsed *nrf2* expression was also observed in the data used in this study.^28^ Pulsed and sustained RNA dynamics can be described with a threshold activation model:
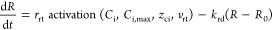
1
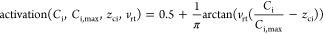
2

The model describes the relative differential transcription of RNA, denoted *R*, as a zero-order kinetic^32,33^ process with a constant transcription rate *r*_rt_, activated when internal concentrations exceed a threshold *z*_ci_ (Figure 1). The slope of the dimensionless activation function (eq 2) is controlled by parameter *v*_rt_. The activation can be any sigmoid function between 0 and 1 if scaled internal concentrations are used. Here, the internal concentration *C*_i_ is scaled with the maximum observed internal concentration over all experiments *C*_i,max_, in order to increase the numerical stability of the function and harmonize the scales of the *v*_rt_ parameter (further details in S1).

*R* degradation follows first-order kinetics^34,35^ with the rate constant *k*_rd_ when the initial RNA concentration *R*_0_ is exceeded. Here we assume *R*_0_ = 1, which makes the modeled quantity identical to the measured fold change. Note that this only applies when the baseline RNA expression is assumed constant, which it likely is not in a developing organism. For a deeper treatment of the relationship between differential RNA expression and measured fold change values, refer to Section S2. Eq 1 results in sustained gene expression, when *C*_i_ never falls below the threshold and it results in pulsed expression when the internal *C*_i_ only transiently exceeds the internal concentration threshold.

#### Uptake and Metabolization

*nrf2* activation is linked to antioxidant response element (ARE) translation, especially, when activated via the AhR pathway, Nrf2 activates metabolization proteins *P* that are involved in degrading the chemicals that activated the response. Because no direct measurements of these proteins are available, the scale of this variable cannot be estimated from the data. However, from measurements of the RNA dynamics and internal concentration dynamics, information on the dynamics of *P* can be obtained, described by the protein degradation rate constant *k*_p_.
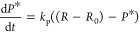
3

P* is the scaled protein concentration that has the same unit as *R* and is proportional to the true (unmeasured) protein concentration *P*. Eq 3 accounts for metabolization reactions that persist after transient gene-regulation pulses, based on the higher stability of proteins with half-lives between 20–46 h^36^ over *nrf2* transcripts with approximated half-lives of 20 min.^26^ A detailed derivation of eq 3 is provided in Section S3 from the full protein dynamics model eq S5, which can be used if actual protein measurements are available.

Since also metabolite concentrations were unavailable, we assume a simplified metabolization process that is described by the concentrations of internal concentration *C*_i_, scaled (metabolizing) protein P* concentration, and a scaled metabolization rate constant *k*_m_, leading to an overall equation for the internal concentration
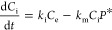
4

The latter term can be understood as Michaelis–Menten enzyme kinetics for relatively low substrate concentrations *C*_i_. Passive detoxification, independent of the P* level, is not considered in this model.

#### Survival

The survival probability *S* is modeled according to the stochastic death assumption of the GUTS framework,^18,37^ where the hazard is approximated by *nrf2* fold-change.
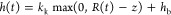
5
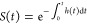
6

#### Error Models

As error models, we use log-normal distributions centered around the predictions of the *nrf2* and internal concentration measurements to account for the fact that these values are constrained to the positive scale (eq S10). For survival data, a conditional binomial model was used (eq S12), and is equivalent to the multinomial model for survival, which is the suggested likelihood function for estimating parameters for survival of small sampling groups with repeated observations over time.^16,37^ The joint likelihood of all observations used in any model is computed with eq S13, assuming that the errors (residuals) are independent of time and measurement variables. Note that the Bayesian credible intervals (BCI) in the visualizations consider only the posterior distribution of the deterministic model parameters. Therefore, they only reflect the expected trajectory of the deterministic model and its parametric uncertainty but not the residual (predictive) error.

#### Standard GUTS Models

The GUTS-RNA-pulse model was compared to additional GUTS model variants: GUTS-reduced (fitted only to survival data, S11.1), GUTS-scaled-damage (fitted to survival data and internal concentrations S10.1), full GUTS (fitted to survival, internal concentration, and *nrf2* fold-change data, S9.1, referred to henceforth as GUTS-RNA). These models have been described in detail^18,37^ and will consequently not be further detailed in this study.

### Data Description

#### *nrf2* Data

This study utilizes a published data set (https://academic.oup.com/gigascience/article/8/6/giz057/5505355) of gene-expression time series of ZFE exposed to diuron, diclofenac, and naproxen from 24 h post fertilization (hpf) to 120 hpf.^28^ The details on the underlying methods are available in the publication's Supporting Information.

#### *C*_ext_, *C*_int_, and Survival Data

To achieve the goals of this work, the *nrf2*-data set has been complemented by time-resolved external concentration measurements (*C*_ext_), internal concentration measurements (*C*_int_), and apical effect observations. The data originate from a series of laboratory experiments conducted at the UFZ in Leipzig and are described in the following.

##### Test Substances

Active substances in the exposure experiments were diclofenac sodium salt (CAS: 15307-79-6, purity: n/a, batch: BCBP9916 V, supplier: Sigma), diuron (CAS: 330-54-1, purity: 99.6%, batch: SZBB265XV) and naproxen sodium salt (CAS: 26159-34-2, purity: 98–102%, batch: MKBV4690 V, supplier: Sigma). Exposure solutions were prepared freshly (<24 h) for each experiment by dissolving preweighed amounts of diclofenac or naproxen in ISO-H2O (ISO 7346-3: 79.99 mM CaCl_2_·2H_2_O, 20.00 mM MgSO_4_·7H_2_O, 30.83 mM NaHCO_3_, 3.09 mM KCl; pH 7.4, oxygenized). In the case of diuron, exposure solutions were prepared from stocks with the substance dissolved in methanol (CAS: 67-56-1; purity: 100%, batch: n/a, supplier: J. T. Baker). The final solvent concentration in exposures and the respective controls was 0.1%. Test concentrations (Table S1) were prepared in serial dilutions shortly before exposure initiation.

##### Zebrafish Handling and Exposure

For all experiments, eggs from zebrafish of the OBI/WIK UFZ strain were reared under constant conditions throughout all included experiments (carbon-filtered tap water, 26 °C, continuous aeration, 14:10 h light:dark cycle). Within 30 min after spawning, eggs were collected. Exclusively, fertilized and undamaged eggs in the 4–32 cell stage were selected for further processing. To ensure comparability to the gene-expression data set,^28^ ZFE was incubated until exposure at 24 hpf. The following day, coagulated, damaged, or developmentally delayed ZFE was discarded. Healthy ZFE was exposed by transferring three ZFEs with 50 μL of ISO-H_2_O into a 7.5 mL glass vial prefilled with 6 mL of exposure solution or control medium. Six replicates were used in negative controls, and for each substance concentration, ZFE was exposed in triplicates, with each replicate containing three organisms. Presorted as well as exposed ZFE were incubated in a climate chamber (Vötsch 1514, Vötsch Industrietechnik GmbH) at 26 °C with a 12:12 h light:dark cycle on a shaker (Edmund Bühler SM-30 Control) with 75 rpm. Apical effects were observed under a stereo light microscope at 24, 48, 72, and 96 hpe. Based on the results of acute toxicity tests, ZFE was exposed in additional sets of experiments at a concentration around the LC_25_ derived for each test substance to determine internal concentrations (*C*_int_). Contrary to acute exposures, each replicate consisting of 10 ZFE was exposed in either 20 mL glass vials containing 18 mL exposure solution or control medium or 7.5 mL glass vials containing 6 mL, depending on the experiment (for details see Table S1).

##### Sampling, Preparation, Extraction, and Measurement of Internal and External Concentrations

After 1.5, 3, 6, 8, 10, 12, 24, 36, 48, 60, 72, 84, and 96 hpe, the replicates were pooled, and samples for analysis of exposure concentrations were taken. Due to the large number of experiments included in this data set, not all treatments included the same number of sampling times. Dead or manually damaged organisms were discarded, and from the remaining, 20 ZFE were randomly selected and transferred into an MP-tube. In the case of internal concentration samples, ZFE were pipetted dry, rinsed once with 1 mL of ISO-H_2_O, and pipetted dry again. All samples were immediately frozen in liquid N2 and stored at −20 °C (*C*_int_) or −80 °C (gene expression) until further processing. External concentration and homogenized internal concentration samples were measured with a liquid chromatography-high resolution mass spectrometry (LC-HRMS) system; for details, see Section S5.

### Parameter Estimation

One of the challenges for developing integrated TKTD models is the structure of the experimental data. Although the work aims to make assessments of the biological processes within one organism, the experimental data are fragmented across many organisms, due to experimental necessity. Thus, fitting the model on individual replicates is simply not possible, because replicates often include only one, sometimes two endpoints. In this work, this data set consisted of 202 treatments, 941 observations distributed over 23 time points and 3 endpoints (Table S1), where the number of observations is moderately unbalanced (internal concentrations: *n* = 539, *nrf2*: *n* = 169, survival: *n* = 233). The number of missing information is several times larger than the number of observations 202 × 23 × 3–941 = 12,997. To overcome this challenge, data sets from numerous biological experiments need to be combined together into a single large data set, which is used to estimate the parameters of the described models. In this work, Diffrax^38^ was used to efficiently evaluate the deterministic model and provide gradients (≈40 ms for solving the ODEs of all 202 experiments). Numpyro^39−41^ was chosen as a probabilistic programming language (PPL) to estimate the distributions of the model parameters with Bayesian methods. Uninformative log-normal priors with 2 standard deviations on the log scale (Section S4.4) were used for the parameter estimation. The state-of-the-art gradient-based Markov-chain Monte Carlo (MCMC) sampler, NUTS,^42^ was used to infer the parameters for the GUTS-reduced model, and stochastic variational inference (SVI)^43^ was used for all other models which were computationally more demanding. The exact details are described in Section S7.1. Model development and parameter estimation were carried out with the modeling platform pymob (https://github.com/flo-schu/pymob), which allows seamless switching between parameter optimization/estimation algorithms. To assess parameter uncertainty and identify identifiability issues, 100 estimations were started with initial parameters drawn from a uniform interval from −1 to 1, which were subsequently transformed to the log-normal scales priors of the parameter distributions. The exact algorithm is described in Section S7.3. The posterior uncertainty in the model parameters is shown with Bayesian credible intervals (BCI, Section S4.5).

## Results

### Using Time-Resolved *nrf2* Data in Combination with a Reversible Damage Dynamic in TKTD Models Is Possible and Can Correctly Describe the Dynamics of Survival

In the first step of this work, four different models were fitted on the available data on a per-substance basis (GUTS-reduced, GUTS-scaled-damage, GUTS-RNA, and GUTS-RNA-pulse). The GUTS-scaled-damage model serves as a baseline and was solely fitted on internal concentration and survival data.

**Figure 2:**
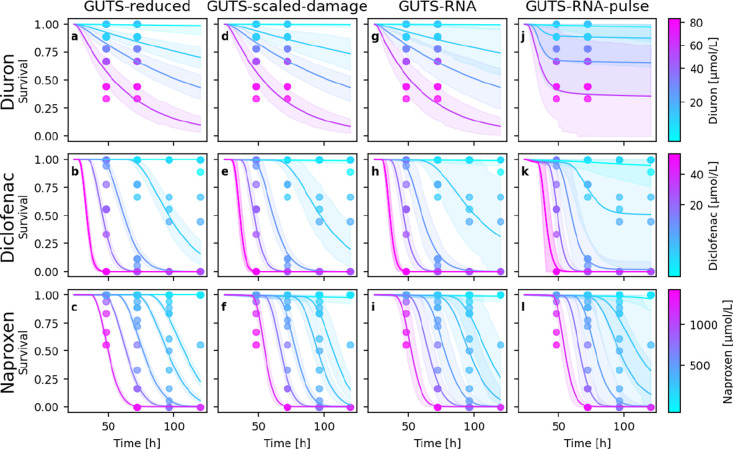
Survival probability estimates and their Bayesian credible intervals (BCI) under exposure to different chemicals (rows) for the different GUTS model flavors (columns) over time, where *t* = 0 is the moment of fertilization of the egg. (a–c) GUTS-reduced model fits survival *S* data only. (d–f) GUTS-scaled-damage mode fits internal concentrations *C*_i_ and *S*. GUTS-RNA (full GUTS) model fits on *C*_i_, *D* with *nrf2* expression as a damage proxy and *S*. (j–l) GUTS-RNA-pulse model fits on *C*_i_, *D* with *nrf2* expression as damage proxy and *S*. The rows display different substances (diclofenac, diuron, and naproxen).

Survival after exposure to diclofenac and naproxen can be successfully modeled with the GUTS-scaled damage (Figure 2e,f) and the GUTS-reduced (Figure 2b,c) model. However, both models are unable to fit constant survival over time at continuous exposure to diuron (Figure 2a,d).

The GUTS-RNA model uses the fold-change values of *nrf2* gene expression as a proxy for the damage state. This integration does not affect the matching of modeled and observed survival for the different substances (Figure 2g,h), but it reduces the variability in the killing rate *k*_k_ and threshold *z* parameters of the stochastic death model across substances (Table S3). In this sense, the integration of the *nrf2* expression data into a TKTD model is already successful. In general, the large variability in observed data, introduced by processing over 200 treatments in one model, is reflected in the large Bayesian credible intervals (BCI) of the model fits. Contrary to intuition, the BCIs further increased with an increasing number of qualitatively different observations (survival, internal concentrations, gene expression) in the model.

### Modeling Damage as a Reversible Process Allows Describing Constant Survival at Continuous Exposure

The implemented RNA–protein dynamic (eqs 1 and 3) approximates key biological processes in the stress response. Only the GUTS-RNA-pulse can accurately reproduce the survival dynamics in all three investigated substances (Figure 2j–l). This can be explained by the ability of the GUTS-RNA-pulse approach to model transient damage pulses, meaning reversible damage at constant exposure. The improved fit of the model comes at the cost of an increase in the number of parameters and wider posterior parameter distributions, which propagate to wider BCIs. While the full GUTS-RNA model needs 7 parameters (excluding the error parameters), the GUTS-RNA-pulse model requires 10 parameters. However, those parameters are connected to explicit biological meanings (Table 1).

**Figure 3:**
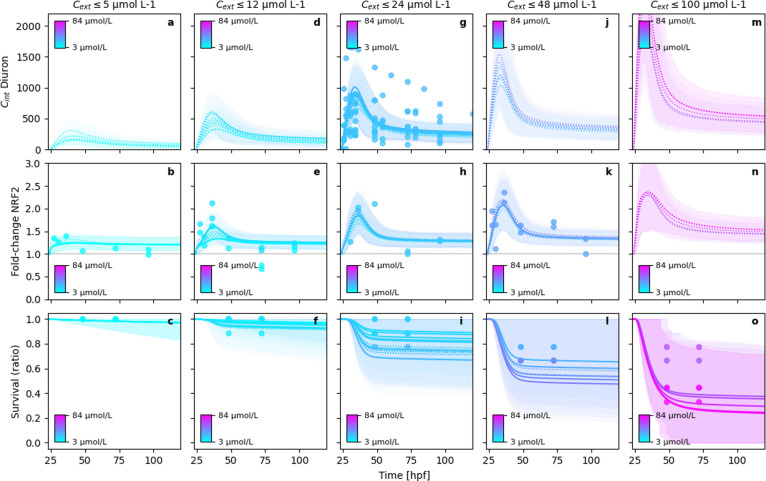
Exemplary model fits and 95%-BCIs of the GUTS-RNA-pulse model for diuron exposure. To improve the readability of the figure, columns distribute the modeled experimental data into concentration classes. The solid lines are the mean posterior estimates of the end points over time and the dotted lines are those estimates where no data were available. The shaded areas are the 95%-BCIs and indicate the posterior density intervals containing 95% probability of the posterior predictions. Note that the residual error in the observations is not included in the BCIs shown in the figures.

The value of the added reversible *nrf2* dynamics is showing particularly well in the case of the diuron exposure (Figure 3). Here, the dynamics of *nrf2* can be described very well over a wide range of concentrations. In addition, the extrapolation of *nrf2* predictions to concentration ranges that were not measured looks promising, as the model limits *nrf2* expression even at high concentrations to reasonable ranges. This behavior of the GUTS-RNA-pulse model is similar for all investigated compounds (Figures S4 and S5). In contrast, at high concentrations, the GUTS-RNA model predicts very high *nrf2* expression (Figures S11–S13).

In addition to the improved description of the *nrf2* dynamics, also the internal concentration dynamics of diuron (Figure 3a,d,g,j,m) and naproxen (Figure S5a,d,g,j,m,p) are more reasonably described by the RNA-pulse model compared to other GUTS variants (Figures S11–S19). This supports the assumption that active metabolization of chemicals under constant exposure has likely been a relevant process in these experiments, Figure 2.

Evidently, the GUTS-RNA-pulse model is also not complete. Despite the improved description of survival and internal concentration, the metabolization kinetics of diuron are overestimated (Figure 3a,d,g,j,m). This can be explained by the very abrupt change in the accumulation rate of diuron in the ZFE. Coupled with the described signaling pathway *C*_i_^↑^ → *nrf2* → P* → *C*_i_^↓^, only a very fast metabolization rate can compensate for the induced time lag between the arrival of the compound in the internal compartment and the onset of metabolization. This leads to a metabolization overshoot, which explains the modeled kinetics.

Also for diclofenac, observed *nrf2* and *C*_i_ dynamics are very complex (Figure S4). If individual concentration trajectories are inspected, multiple enrichment phases are visible: fast initial uptake is followed by slowed uptake, and followed by reduction of the internal concentration. Such dynamics are not yet possible to be modeled with a simple RNA pulse model. Obviously, the true dynamics of the signaling pathways of the stress response are much more complicated and have been modeled with more than 30 ODE terms.^44^ Note also that the higher contribution of *C*_i_ to the joint likelihood function due to an increased number of data points (*n* = 539) over nrf2 (*n* = 169) and survival (*n* = 233) and scale effects of higher concentrations in the log-normal likelihood function may have induced slight biases in the model fits.

Despite these limitations, the proposed GUTS-RNA-pulse model has several advantages. First of all, using the expression of *nrf2* as a proxy for toxicodynamic damage in the TKTD-RNA-pulse model successfully linked the internal concentration dynamic and survival dynamic. In addition, the model closely matched the dynamic of *nrf2* expression in the case of diuron. This suggests that gene expression data can be integrated into TKTD models to approximate toxicodynamic damage; therefore, we maintain hypothesis 1: (“Toxicodynamic damage can be approximated by gene-expression data ···”). In addition, it provides a possible solution to the problem of modeling reversible damage and predicting constant survival at continuous exposure to toxic chemicals on the level of the individual. Furthermore, the RNA-protein dynamics eqs 1 and 3 should be independent of the exposed substance and with them the coupled stochastic death model.

### Survival Dynamics Can Not Solely Be Predicted by an *nrf2* Damage Proxy

After having established the integration of time-resolved *nrf2* data into a TKTD model, we go on to test the hypothesis that the level of *nrf2* expression predicts lethality in zebrafish embryos in a general way, independent of the specific substance. For this, we take the GUTS-RNA-pulse model and allow the sharing of 7 parameters among the 3 substances in the parameter estimation, leaving only the parameters controlling uptake *k*_i_, metabolization *k*_m,_ and the gene-expression activation threshold *z*_ci_ as substance-specific parameters. Hereafter, the parameter-sharing model will be referred to as the substance-independent model. The substance-independent model has 7 + 3 × 3 = 16 parameters (excluding error parameters), compared to the substance-specific models (Figures 3, S8, and S9), which together have 3 × 10 = 30 parameters. If *nrf2* was a valid predictor (precursor) for lethality and the model dynamic was accurately described, the model with fewer parameters should describe the lethality equally well.

**Figure 4:**
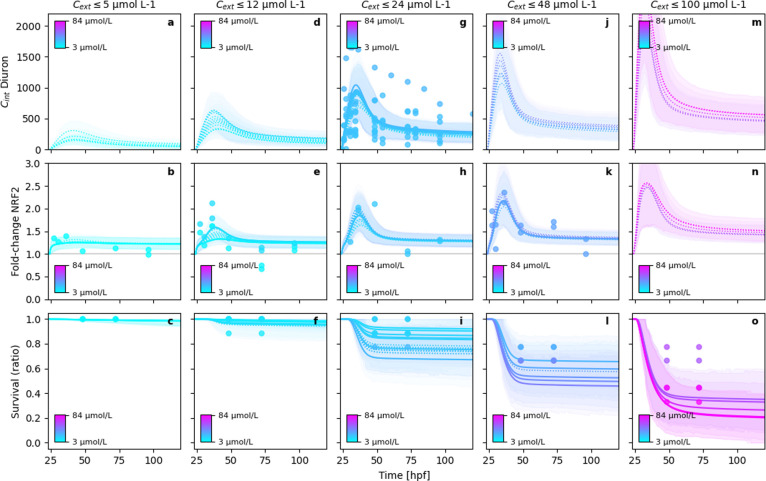
Exemplary model fits and 95%-BCIs of the parameter sharing (substance-independent) GUTS-RNA-pulse model for diuron exposure. In order to improve the readability of the figure, columns distribute the modeled experimental data into concentration classes. The solid lines are the mean posterior estimates of the end points over time and the dotted lines are those estimates where no data were available. The shaded areas are the 95%-BCIs indicate the posterior density intervals containing 95% probability of the posterior predictions. Note that the residual error in the observations is not included in the BCIs shown in the figures.

The substance-independent model indeed delivers accurate model fits for all observed endpoints after diuron exposure (Figure 4). Diclofenac fits of the substance-independent model (Figure S8) are also very similar to the fully substance-specific model, although the predicted *nrf2*-expression pattern is not in exact agreement with the data similar to the fully substance-specific model (Figure S4).

Fits of naproxen data with the substance-independent model indicate that *nrf2* gene expression data is not sufficient to predict lethality in zebrafish embryos (Figure S9). The observed accurate fits for lethality can only be achieved by large deviations of the model fits from internal concentration measurements and *nrf2* expression measurements (Figure S9). This deviation is underlined by the higher total Bayesian information criterion (BIC) value (for all three substances) of the substance-independent model (BIC = 6337) compared to the substance-specific model (BIC = 5940). Overall, the fits of the substance-independent model were surprisingly good, despite having 14 parameters less than the substance-specific model. Nevertheless, our results show that our second hypothesis cannot be confirmed, and *nrf2* gene expression alone is not sufficient to predict lethality with the developed model. *nrf2* is known to play a key role in the induction of stress response, including metabolization and detoxification. The observed data patterns underline this, especially for diclofenac and diuron.

### Sharing Parameters of the RNA–Protein Dynamic between Different Substances Eliminated the Problem of Parameter Identifiability

**Figure 5:**
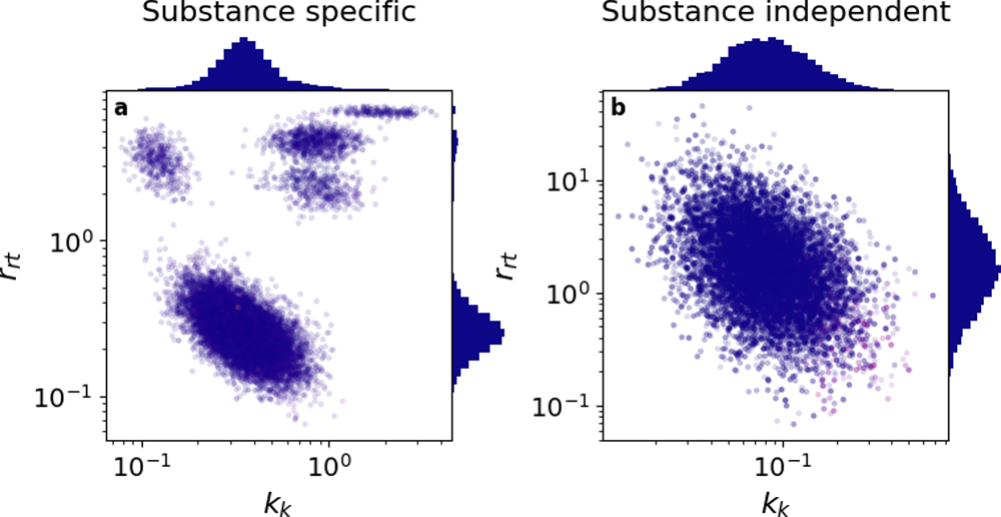
Joint posterior parameter distribution of *k*_k_ and *r*_rt_ for the diclofenac fit. The model converges on multiple clusters of parameter estimates with very similar likelihood. (a) Substance specific model. (b) Substance independent model.

Due to the model complexity, the GUTS-RNA-pulse model suffered from parameter identifiability issues. This is exemplary, shown in Figure 5 a for the case of diclofenac. The parameter clusters have slightly different likelihoods, which originate from the deviation of parameter estimates from the prior probabilities. While these issues could be cured by simplifying the model, they could also be tackled by using more concentrated prior probabilities. The GUTS-RNA-pulse model allows this because here most parameters have very specific biological meanings and could, in theory, be informed by empirical evidence from previous experimental work or expert knowledge.

Here, the problem was solved by making the RNA–protein dynamic substance independent (Figure 5b), reducing the number of parameters from 30 to 16, and consequently the uncertainty in parameter estimates. The parameter inference approach applied in this work joined scattered data sets and extracted information from them, by combining effect measurements from multiple process steps. It would be impossible to fit models separately on individual experiments because the observed endpoints never jointly occur in a single experiment as all material from the exposed organism was consumed for the separate analyses.

## Discussion

### Time Resolved Gene-Expression Data Can Be Integrated into TKTD Models as a Proxy for Toxicodynamic-Damage

The GUTS-RNA-pulse model described in this work delivered high-quality model fits with respect to the endpoint dynamics. We therefore maintain our hypothesis that time-resolved gene expression data can be integrated into TKTD models. While it is generally accepted that increasing the number of observations reduces the parametric uncertainty, in this work we observed the contrary for the successive increase in the number of endpoints added to the model. Each addition added further sources of uncertainty (data variability and model structure) to the model, widening the credible intervals of the model dynamics (and of the parameter estimates) with each additionally included endpoint. What does this mean? On one hand, inaccurate specification of the model structure of the added endpoint can inflate the parametric uncertainty because even partially incorrect models may only be able to cover all data by widening the parameter distributions. In addition, each added endpoint incorporates additional sources of variability (experimental variability, random biological, and measurement errors) into the model. This can, if not properly addressed, also inflate the parametric uncertainty. On the other hand, adding qualitatively different information (as opposed to increasing the quantity) imposes constraints on the model and makes flaws in the model transparent, requiring a critical review of the model’s assumptions and specifications. This facilitates the development of more realistic models with potentially better physical interpretation and predictive capabilities.

Therefore, increasing the number of different end points in a model is challenging, but if carefully done, it will help to better understand processes governing the data and to predict their outcomes.

### Hierarchical Error Modeling for Better Identifiability of Model Parameters

In the present work, we used a complete pooling approach, i.e., fitting the same model on all data, as opposed to using a hierarchical approach^45,46^ where different experiments may have varying parameter estimates. While this complete pooling approach is easier to implement, it has drawbacks. Since the data used in this work originated from multiple experiments, it contains complex nested error structures with 3 layers of errors:

1. Experimental errors, e.g., biological batch differences between experiments, microarray batch effects, or variation in prepared stock solutions.
2. Treatment errors, e.g., pipetting errors.
3. Random (white noise) errors, e.g., measurement errors, or biological variation within the same batch.

These error levels are present in different depths of observations depending on the observed endpoint (repeated observations of the same individual in lethality measurements vs independent measurements of *nrf2* and *C*_i_ data over time).

In the current approach, the same model will be forced on observations from different experiments and treatments; if the initial conditions (i.e., exposure) differ between experiments due to batch errors or experimental handling between experimentation, this will result in fitting a model that has to satisfy heterogeneous data with a single parameter per replicate or experiment. This can lead to biased estimations of model parameters, which can be more severe under nonlinear dynamics.^47^ A better alternative is a partial pooling (hierarchical) approach where group-level means are fitted which themselves serve as priors for the parameter estimates of individual experimental time series.^47^ Finding an accurate representation of the error structure also allows for the use of fixed error parameters. In the current approach, error parameters σ (Table 1) were fitted from the data and included noise from all error sources. However, often knowledge exists about the variability of measurement noise. Overall, a hierarchical approach allows a more exact attribution of errors to different levels of hierarchy and allows the use of fixed error parameters for, e.g., measurement errors, which allows the model to fit closer to the intermediate end points (*C*_i_, *nrf2*) and yield more robust uncertainty estimates for the survival fits.

### *nrf2*’s Role in the Stress Response from a TKTD Perspective

While the biology of the ISR is very complex and involves numerous genes, proteins, and enzymes,^20,21,25,44^ the development of generalized models requires simplifications. In the presented model, we focused on *nrf2* and let it play a double role as a proxy for toxicodynamic damage and a downstream trigger of biotransformation and detoxification. We tested whether *nrf2* is a general predictor of lethality for any of the tested substances and concentrations. With the here described model, this hypothesis could not be confirmed. This answer follows from the deterioration of model fits for internal concentrations and *nrf2* data when using substance-independent parameters for the RNA–protein dynamic eqs 1 and 3. Nevertheless, we found ample evidence that *nrf2* is closely related to the process of activating metabolization and, in that sense, at least indirectly linked to lethality.

By modeling the activation of metabolization by gene transcription and protein synthesis, the phenomenon of reversible damage at constant exposure to toxicants was addressed. Conventionally, uptake and removal processes in TKTD models are directly linked to the external concentration even if external concentrations are propagated through multiple state variables (*C*_i_, *D*). This results in monotonically increasing dynamics at constant concentrations. To model nonmonotone damage dynamics under constant concentrations, internal concentration dynamics or damage dynamics need to be (partially) decoupled from external concentrations. In this work, this was achieved by the activation of metabolization by gene transcription and protein synthesis, resulting in a temporal delay of the metabolization process. Decoupling can also occur through interactions with the dynamics of other compounds,^14^ through interactions with other time-dependent processes, or through temporal variation in the rate constants.

Decreasing internal concentrations before the end of the exposure have been observed in ZFE after exposure to diazinon exposure at 0.4 ppm.^48^ In the same study, exposure to 1/4 of this concentration did not lead to decreasing internal concentrations before the end of the exposure. In a different study, decreasing internal concentrations at constant exposure levels were observed for a variety of compounds such as benzocaine, phenacetin, metribuzin, phenytoin, or valproic acid;^49^ while the size increase of the developing organism could not account for the decrease in internal concentrations. In another exposure study of α-cypermethrin on *Daphnia magna*,^14^ modeling active metabolization led to excellent model fits with constant survival predictions for diuron (Figure 3), which indicates that a threshold model for active metabolization of a compound is indeed relevant. It was previously shown that temporal gene transcription changes of cytochrome P450 enzymes after exposure to benz[α]anthracene follow pulse-like, fluctuating, or monotonically increasing dynamics that were additionally sensitive to the developmental age of ZFE.^50^ Following this, temporally resolved concentrations of metabolites or RNA expression data for metabolic enzymes should be integrated into the proposed model to couple active metabolization with RNA expression by using advanced kinetic models such as, e.g., Michaelis–Menten kinetics. This could significantly improve the model, by considering self-reinforcing feedback loops from toxic metabolites, which allows the modeling of fluctuating RNA expression patterns and multiphase-internal concentration and survival dynamics.

The substance-independent GUTS-RNA-pulse model estimates an RNA decay rate constant of 1.3/*t* (0.1–3.3), translating to a half-life distribution with a mode at 23 min and highly probable values between 0 and 1.5 h (Figure S23). This is in excellent agreement with experimentally established RNA half-lives of 10–20 min.^24,26^ Although protein kinetics were only calibrated indirectly, the estimated protein stability (half-life (mode) ≈ 46 h with wide tails toward longer half-lives, Table S24) agreed with reported ranges of proteins between 20–46 h.^36^ The agreement of fitted RNA half-life and protein half-life with reported literature data indicates that it is possible to use informative priors for RNA decay in future work. The strong differences in molecular kinetics underline the importance that the temporal dimension has to be considered to understand the relationship between transcription and enzyme activity^51^ and diverging measurements.^52^

Adding the *nrf2* expression as mechanistic information to the TKTD model can increase the interpretability of the killing rate parameter: Because *nrf2* appears in the hazard function, the unit of *k*_k_ in an unscaled model version would be L μ mol *nrf2*^–1^ h^–1^. Because Nrf2 is known to induce cell death (apoptosis) when the stress cannot successfully be reduced,^21^ the killing rate parameter can be interpreted as the volume of dead cells per *nrf2* fold-change increase above the threshold per unit time. However, this interpretation is only possible when information on the *nrf2* expression of the untreated organism is included, leading to the replacement of fold-change with μmol L^–1^.

## Outlook

The presented model is far from perfect and is not close to the goal of reaching a predictive risk assessment without the need for animal testing. However, we are very confident to have shown a method that has immense potential to bring mechanistic understanding into risk assessment. We see three major advantages arising from this: First, better extrapolation occurs through the incorporation of further biological processes. Second, exploration of the structural uncertainty of TKTD models through the inclusion of additional biological end points. Third, the ability to formulate experimentally falsifiable hypotheses and identification of clear molecular targets for testing the hypotheses (models) with the goal of successively improving model predictions.

Multiple concrete research directions are envisioned to proceed toward maturing this approach.

1. Deepening the model detail with respect to the biotransformation processes and enzyme kinetics to extend the model with respect to repair and rescue capacity.
2. Improving the error modeling to account for experimental variation and improve uncertainty assessment.
3. Integrate further molecular end points, such as oxidative stress markers,^53^ or whole transcriptome expression data^28^ into the model. However, such a process has high computational demands.
4. Make use of Bayesian parameter inference, by integrating prior knowledge into protein dynamics. It is known that proteins have a significantly larger half-life than RNA (20–46 h).^36^
5. Model sublethal effects, which are readily available for ZFE and can be investigated with the same approach with the potential to reduce animal testing. A respective database and software (INTOB) for systematic and FAIR ZFE phenotype data, including sublethal effects, will be available soon and might provide a valid prerequisite.
6. Integrate molecular information on the untreated organism into the model to move from a scaled model version (relative to the baseline organism) to an untreated organism to account for developmental processes within the lifetime of the zebrafish embryos.
7. Validate the model against other substances in order to build confidence into the model or identify deficiencies.
8. Extend the model to describe different types of typically observed RNA expression patterns such as fast-forward loops, cascades, autoregulation loops, etc.^30^ For instance, Nrf2 may induce KEAP1 expression which might limit uncontrolled *nrf2* expression.^27^

## Supporting Information Available

Code used to generate the results including install and usage instructions are available on https://github.com/flo-schu/tktd_nrf2_zfe/; modeling notebook aligned with TRACE standards is available on https://github.com/flo-schu/tktd_rna_pulse/blob/main/scripts/tktd_rna_3_6c_substance:specific.ipynb; GUTS-RNA-pulse models are available on https://github.com/flo-schu/tktd_rna_pulse; standard GUTS models (reduced, scaled-damage, full GUTS) are available on https://github.com/flo-schu/guts; code for fitting parameters of the model is available on https://github.com/flo-schu/pymob; data sets are available on 10.12751/g-node.l6jqgf (https://gin.g-node.org/flo-schu/tktd_nrf2_zfe__data); additional results for the GUTS-RNA-pulse models are available on https://gin.g-node.org/flo-schu/tktd_rna_pulse__results; and additional results for the standard GUTS models (reduced, scaled-damage, full GUTS) models are available on https://gin.g-node.org/flo-schu/guts__results (PDF)

## Abbreviations

TKTD: toxicokinetic–toxicodynamic
GUTS: general unified threshold model for survival
ISR: integrated stress response
ARE: antioxidant response elements
ZFE: zebrafish embryo
hpe: hours post exposure
hpf: hours post fertilization
PPL: proabilistic programming language
MCMC: Markov–Chain Monte Carlo
SVI: stochastic variational inference
BIC: Bayesian information criterion
