# Supplementary material for: Integrating Time-Resolved nrf2 Gene-Expression Data into a Full GUTS Model as a Proxy for Toxicodynamic Damage in Zebrafish Embryo
Source: Environ Sci Technol. 2024 Dec 4;58(50):21942–53. doi: 10.1021/acs.est.4c06267 (PMC11656706; doi:10.1021/acs.est.4c06267)
Supplement: Supplementary file 1 — es4c06267_si_001.pdf [file es4c06267_si_001.pdf]

Supporting Information for: Integrating time-resolved *nrf2* gene-expression data into a full GUTS model as a proxy for toxicodynamic damage in zebrafish embryo

Florian Schunck[1,*], Bernhard Kodritsch[2], Martin Krauss[2], Wibke Busch[2], Andreas Focks[1]

[1] Osnabrück University, Barbarastr. 12, 49076 Osnabrück, Germany
[2] Helmholtz-Centre for Environmental Research GmbH—UFZ, Permoserstr. 15, 04318 Leipzig, Germany

[*] Corresponding author, florian.schunck@uni-osnabrueck.de

This document contains 24 Figures and 10 Tables on 35 pages.

Contents

S1 Activation function for RNA expression . . . S3
S2 Interpreting fold-change quantities in the context of a dynamic model . . . S3
S3 Scaled protein concentrations . . . S5
S4 Error models . . . S6
S4.1 Likelihood functions of internal concentration and *nrf2* . . . S6
S4.2 Likelihood function of survival . . . S6
S4.3 Joint Likelihood . . . S6
S4.4 Model priors . . . S6
S4.5 Bayesian credible intervals (BCI) . . . S7
S5 LC/MS measurement method for internal and external concentrations . . . S7
S6 Experiments . . . S7
S7 Parameter estimation . . . S11
S7.1 Bayesian parameter inference . . . S11
S7.2 Leveraging modern probabilistic programming languages (PPL) to solve the computational challenges of the 'omics integration into TKTD models . . . S11
S7.3 Parameter analysis algorithm . . . S12
S8 GUTS-RNA-pulse model . . . S14
S8.1 Model description of the GUTS-RNA-pulse model (substance specific and substance independent) . . . S14
S8.2 Model fits for GUTS-RNA-pulse . . . S15
S8.3 Model fits for GUTS-RNA-pulse model with parameter sharing for the RNA and protein modules . . . S19
S9 GUTS-RNA model . . . S22
S9.1 Model description of the GUTS-RNA model . . . S22
S9.2 Model fits for GUTS-RNA . . . S23
S10 GUTS-scaled-damage model . . . S26
S10.1 Model description of the GUTS-scaled-damage model . . . S26
S10.2 Model fits for GUTS-scaled-damage . . . S27
S11 GUTS-reduced model . . . S30
S11.1 Model description of the GUTS-reduced model . . . S30

S11.2 Model fits for GUTS-reduced S31
S12 Estimated half-life of nrf2 . . . . S33
S13 Estimated half-life of proteins S33

List of Figures

Figure S1 Panel 1: Control (untreated) RNA expression rate and the treatment RNA expression rate as the combined signal of effect and control. Panel 2: true differential expression and measured differential expression. Panel 3: C_t values over time of control and treatment. Panel 4: Modelled fold-change and measured fold change S4
Figure S2 Left: Time to reach steady state concentration for a one compartment model such as Eq. S5 for different rate constant. k_1 is the synthesis rate and k_2 is the degradation rate (k_p in the case of the RNA pulse model). It is evident, that k_1 regulates the scale of the process, whereas k_2 regulates the response time of the process. Right: Using a scaled variant which still fits the degradation rate, will provide information on the model dynamic but not on the scale of the variable. The red line dashed line is the dynamic of the 2 parameter model scaled by the k_1/k_2 ratio and matches the dynamic of the 1-parameter model. S5
Figure S3 Posterior estimates and 95%-BCIs of the GUTS-RNA-pulse model for diuron . S15
Figure S4 Posterior estimates and 95%-BCIs of the GUTS-RNA-pulse model for diclofenac S16
Figure S5 Posterior estimates and 95%-BCIs of the GUTS-RNA-pulse model for naproxen S16
Figure S6 Parameter estimates of the GUTS-RNA-pulse model with substance specific parameters. S17
Figure S7 Posterior estimates and 95%-BCIs of diuron for the parameter sharing GUTS-RNA-pulse model. S19
Figure S8 Posterior estimates and 95%-BCIs of diclofenac for the parameter sharing GUTS-RNA-pulse model. S19
Figure S9 Posterior estimates and 95%-BCIs of naproxen for the parameter sharing GUTS-RNA-pulse model. S19
Figure S10 Parameter estimates of the GUTS-RNA-pulse model with substance-independent parameters for the RNA and protein dynamics. S20
Figure S11 Posterior estimates and 95%-BCIs of diuron for GUTS-RNA model. S23
Figure S12 Posterior estimates and 95%-BCIs of diclofenac for GUTS-RNA model. S23
Figure S13 Posterior estimates and 95%-BCIs of naproxen for GUTS-RNA model. S23
Figure S14 Parameter estimates of the GUTS-RNA model. S24
Figure S15 Posterior estimates and 95%-BCIs of diuron for GUTS-scaled damage model. . S27
Figure S16 Posterior estimates and 95%-BCIs of diclofenac for GUTS-scaled damage model. S27
Figure S17 Posterior estimates and 95%-BCIs of naproxen for GUTS-scaled damage model. S27
Figure S18 Parameter estimates of the GUTS-scaled-damage model with substance specific parameters. S28
Figure S19 Posterior estimates and 95%-BCIs of naproxen for GUTS-reduced damage model.S31
Figure S20 Posterior estimates and 95%-BCIs of diclofenac for GUTS-reduced damage model.S31
Figure S21 Posterior estimates and 95%-BCIs of diuron for GUTS-reduced damage model. S31
Figure S22 Parameter estimates of the GUTS-reduced model with substance specific parameters.S32
Figure S23 Estimated half-life of RNA expression from the r_{rd} parameter. A simple exponential decay model was assumed to estimate the half life. And a log-normal probability distribution was fitted to estimate the distribution of half-life times. S33
Figure S24 Estimated half-life of proteins from the r_p parameter. A simple exponential decay model was assumed to estimate the half-life. And a log-normal probability distribution was fitted to estimate the distribution of half-life times. S34

List of Tables

Table S1 Included experiment in the present study. C_{ext} refers to the nominal external concentration ($\mu mol\ L^-1$). V_{expo} is the volume of the exposure solution in ml. N_{ZFE} is the number of zebrafish embryos used in a single experimental replicate. N_{Trt} is the number of treatments applied for the given exposure concentration.N_{Obs} refers to the cumulative number of observations for all replicates and observation times. S7
Table S2 TKTD state variables and parameters used in the GUTS-RNA-pulse model. The column "Assumed substance independence" indicates whether a parameter is supposed to be shared for multiple substances. S15
Table S3 Parameter estimates and posterior highest densitiy intervals (HDI) of the substance specific GUTS-RNA-pulse model. The HDI contains 94% of the probable parameter values given the data. fc = fold-change (*nrf2*). S18
Table S4 Parameter estimates and posterior highest densitiy intervals (HDI) of the GUTS-RNA-pulse model with a substance independent RNA protein module. Parameters which share information between substances are given in the form (mean (3% hdi–97% hdi)). Parameter sharing reduces the number of parameters for all 3 substances from 30 to 18. fc = fold-change (*nrf2*). S21
Table S5 TKTD Parameters used in the GUTS-RNA model. S22
Table S6 Parameter estimates and posterior highest density intervals (HDI) of the GUTS-RNA model. The HDI contains 94% of the probable parameter values given the data. fc = fold-change (*nrf2*). S25
Table S7 TKTD Parameters used in the GUTS-scaled-damage model. S26
Table S8 Parameter estimates and posterior highest densitiy intervals (HDI) of the GUTS-scaled-damage model. The HDI contains 94% of the probable parameter values given the data. S29
Table S9 TKTD state variables and parameters used in the GUTS-reduced model. S30
Table S10 Parameter estimates and posterior highest densitiy intervals (HDI) of the GUTS-reduced model. The HDI contains 94% of the probable parameter values given the data. S33

S1 Activation function for RNA expression

$$activation(C_i,\ C_{i,max},\ z_{ci},\ v_{rt}) = 0.5 + \frac{1}{\pi}\ arctan(v_{rt}\ (\frac{C_i}{C_{i,max}} - z_{ci})) \qquad \text{(Eq. S1)}$$

which is numerically stable at high differences between C_i and the threshold z_{ci}, in contrast to the conventionally used logistic function. For the activation calculation, C_i was scaled by the maximum internal concentration $C_{i,\ max}$, in order to make threshold and slope parameters comparable across substances. In addition, this approach makes it also easier to use other activation functions like the logistic function.

S2 Interpreting fold-change quantities in the context of a dynamic model

Gene expression measurements in ZFE are usually based on calculating the differential expression (fold-change) of the treatment to the baseline and are typically reported as fold-change or log fold-change. The transformation of gene expression data (e.g. Δ, fold-change) has different pros and cons. The true effect is the effect that is easiest to model as it requires the least amount of assumptions, since it models only the effect, which should follow a relatively simple scheme [1, 2].

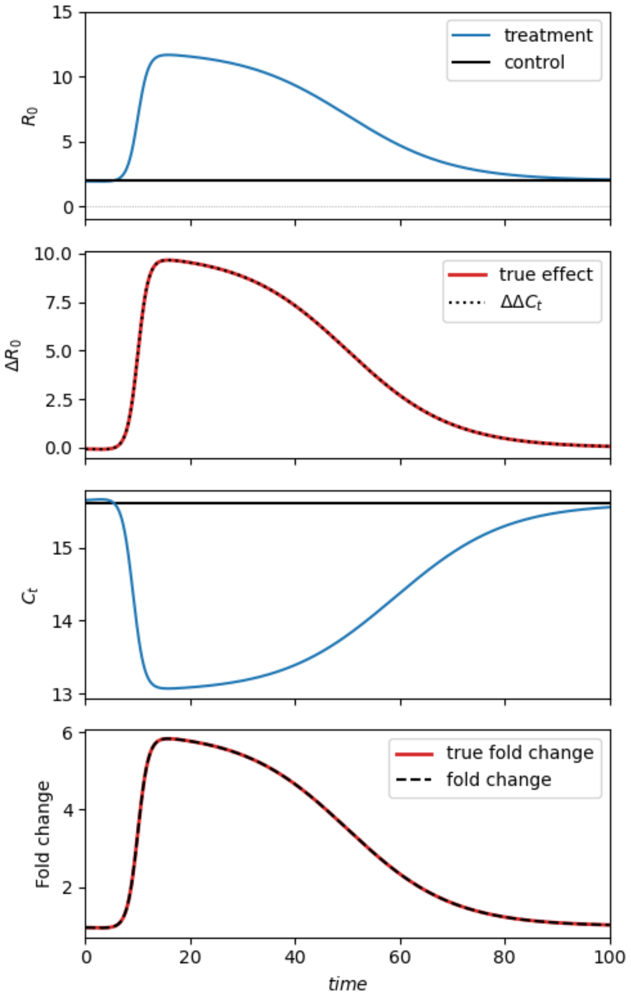

Figure S1. Panel 1: Control (untreated) RNA expression rate and the treatment RNA expression rate as the combined signal of effect and control. Panel 2: true differential expression and measured differential expression. Panel 3: C_t values over time of control and treatment. Panel 4: Modelled fold-change and measured fold change

The disadvantage of using fold-change data is that they are only proportional to the true effect if not scaled by measurement specific information such as the **threshold** value of a qPCR analysis at which the C_t signal is obtained. The comparison of modeled Eq. S2 and measured data Eq. S3 shows that the measured fold change is equal to the true (modeled) fold change (Fig. S1).

$$\text{true fold change} = \frac{R_{\text{treatment}}}{R_{\text{control}}} \tag{Eq. S2}$$

$$\text{measured fold change} = 2^{-(C_t^{\text{treatment}} - C_t^{\text{control}})} \tag{Eq. S3}$$

The major disadvantage of using fold-change data is that a baseline has to be modeled implicitly if the true signal should be modeled recovered. Of course simple baseline models can be used such as a constant signal, increasing baseline (linear), step, ... In addition the baseline could be modeled as a stochastic function, such as a random walk where information from the dynamic of the baseline could come from the raw data of the controls.

If the baseline RNA expression is modeled explicitly, but data are only available as fold change, we recommend rescaling the RNA expression data according to Eq. S4.

$$R_{\text{fc}}(t) = \frac{R(t) + R_{\text{control}}(t)}{R_{\text{control}}(t)} \tag{Eq. S4}$$

S3 Scaled protein concentrations

The full process of the protein dynamic would be at least a 2-parameter differential equation assuming the quantity of proteins are driven by a balance between protein synthesis and protein degradation:

$$\frac{dP}{dt} = k_{ps}\ (R - R_0) - k_p\ P \qquad \text{(Eq. S5)}$$

where k_{ps} describes the protein synthesis rate constant in μmol L^{-1} h^{-1} and k_p describes the protein degradation rate constant in h^{-1}. However, such an equation would require knowledge of the protein levels inside the organism. Since it is not available in this work, we resort to a scaled version of the equation. Along the reasoning of [3, 4], an alternative formulation of Eq. S5 is

$$\frac{dP}{dt} = k_p(\frac{k_{ps}}{k_p}(R - R_0) - P) \qquad \text{(Eq. S6)}$$

In one compartment models the steady state concentration of a quantity can be determined as the ratio between two rate constants. This yields the partitioning coefficient $K_{PR} = k_{ps}/k_p$.

$$\frac{dP}{dt} = k_p(K_{PR}\ (R - R_0) - P) \qquad \text{(Eq. S7)}$$

Dividing both sides of Eq. S7 by the partitioning coefficient leads to

$$\frac{dP/K_{PR}}{dt} = k_p((R - R_0) - \frac{P}{K_{PR}}) \qquad \text{(Eq. S8)}$$

Finally, defining $P^* = \frac{P}{K_{PR}}$ leads to

$$\frac{dP^*}{dt} = k_p((R - R_0) - P^*) \qquad \text{(Eq. S9)}$$

In Eq. S9 P^* is the scaled protein concentration which is proportional to the true protein concentration P, but has the same units as R. As the protein degradation rate is still estimated from the data, the time to reach steady-state will still be estimated, but the scale of the variable is unknown.

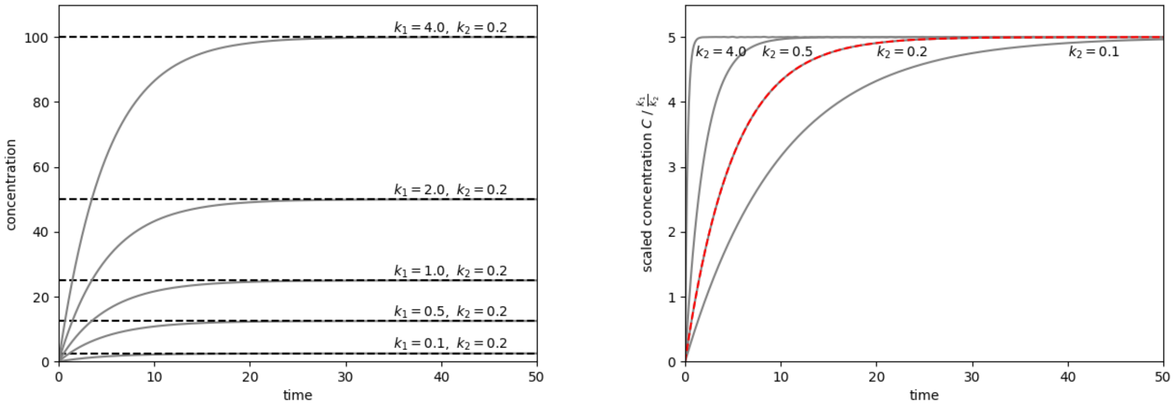

Figure S2. Left: Time to reach steady state concentration for a one compartment model such as Eq. S5 for different rate constant. k_1 is the synthesis rate and k_2 is the degradation rate (k_p in the case of the RNA pulse model). It is evident, that k_1 regulates the scale of the process, whereas k_2 regulates the response time of the process. Right: Using a scaled variant which still fits the degradation rate, will provide information on the model dynamic but not on the scale of the variable. The red line dashed line is the dynamic of the 2 parameter model scaled by the k_1/k_2 ratio and matches the dynamic of the 1-parameter model.

S4 Error models

S4.1 Likelihood functions of internal concentration and *nrf2*

The variables internal concentration C_i and *nrf2* R are considered to follow a lognormal probability distribution, where C_i is exemplarily shown and takes the same form for R.

$$C^{obs}_{i,j}(t) \sim \text{LogNormal}\left(\text{loc} = \log(C_{i,j}(t)),\ \text{scale} = \sigma_{ci}\right) \quad \text{(Eq. S10)}$$

$$\sigma_{ci} \sim \text{HalfNormal}(\text{scale} = 5) \quad \text{(Eq. S11)}$$

where j is the replicate id and t is a time t of all sampling times of the variable and replicate respectively, and t_0 is the starting time of the experiment (24 h in this work). $C_{i,j}(t)$ is the temporal trajectory of $C_i(t)$ is defined in Eq. 4 for the replicate j specific treatment conditions (i.e. exposure concentration).

S4.2 Likelihood function of survival

Survival is considered to follow a conditional binomial probability distribution. It is a binomial probability distribution, where the probability p to survive until a time t is corrected by the probability to have survived until a previous time interval t_{-1}, here the end of a previous observation process.

$$S^{obs}_j(t) \sim \text{Binomial}\left(\text{prob} = \frac{S_j(t)}{S_j(t_{-1})}, n = S^{obs}_j(t_{-1})\right) \quad \text{(Eq. S12)}$$

$S_j(t)$ is the survival probability defined in Eq. 6 for the replicate j specific treatment conditions (i.e. exposure concentration). $S^{obs}_j(t)$ is the number of survivors at time t.

S4.3 Joint Likelihood

In this work errors (residuals) ϵ_{ci}, ϵ_{nrf2} and ϵ_S are assumed to be statistically independent, because the deterministic model described in Eqs. 1–6 is assumed to capture the dependence between the variables appropriately. While the described processes probably include other sources of co-variation between the measured variables, assuming independence between the errors is a first approximation sufficient for a proof of principle. Therefore the joint likelihood is

$$\mathcal{L}(\theta \mid y) = \prod_v^V \prod_t^{T_v} p\left(y_{v,t} \mid f_v(t, \theta_v),\ \sigma_v\right) \quad \text{(Eq. S13)}$$

where v denotes a variable (C_i, R, S) and V is the set of variables present in the respective model (e.g. the reduced GUTS model is only calibrated to S). T_v are the timepoints t at which observations y_v were made for a respective state variable. Note that the individual likelihoods were not weighted to the relative number of observations of the variable.

S4.4 Model priors

Uninformative priors were used for the model. For standard deviations of the lognormal residual error distributions, half-normal distributions with a scale of 5 were used, translating to a very wide standard deviation of 5 on the log scale. For the deterministic model parameters uninformative lognormal priors were used with 2 standard deviations on the log scale around the mode of the distribution. The modes of the prior distributions were manually set so that prior predictions contained all data by wide margins.

The detailed priors (and probability models) used in the work can be reviewed in the following files:

1. (TKTD-RNA-pulse) `https://github.com/flo-schu/tktd_rna_pulse/blob/main/prob.py`
2. (GUTS) `https://github.com/flo-schu/guts/blob/main/prob.py`

S4.5 Bayesian credible intervals (BCI)

The posterior uncertainty into the model parameters is shown with Bayesian credible intervals (BCI). To compute BCIs, the shape of the parameter distributions is propagated to the deterministic model trajectories (C_i, R, S) by drawing samples from the posterior parameter distributions and computing the deterministic model solutions for each joint parameter sample from the posterior. From these samples, 95% highest posterior density intervals (HPDI) of the trajectories are computed. A HPDI is the narrowest interval that contains 95% of the probability mass and is a Bayesian credible interval (BCI). HPDIs are slightly different from equal tail intervals, because equal tail intervals are not necessarily the narrowest intervals, and may, in rare cases, not contain the mode of the distribution.

S5 LC/MS measurement method for internal and external concentrations

Diuron, diclofenac and naproxen were analysed by liquid chromatography-high resolution mass spectrometry (LC-HRMS) using a Ultimate 3000 LC system (Thermo) coupled to a Thermo LTQ Orbitrap XL We used a Kinetex Core-Shell C18 column (50 mm × 2.1 mm; 2.6 µm particle size; Phenomenex) and a gradient elution was carried out with a flow rate of 0.3 mL/min with water (A) and methanol (B) both containing 0.1% formic acid. The initial content of 20% B was linearly increased after 0.5 minutes to 100 % B within 5.5 minutes. B was maintained at 100% for 8 minutes followed by a re-equilibration for 5 minutes. The injection volume was 5 µL and the column was maintained at 40°C. A heated electrospray ionisation source was used in positive mode. Full scan spectra were acquired in centroid mode in a range of 80 to 600 m/z at a nominal resolving power of 30,000 referenced to m/z 400. A mass accuracy $<$ 7 ppm was assured over the whole mass range by external mass calibration using a calibration solution for the range from 138 to 1721 m/z.

S6 Experiments

Table S1. Included experiment in the present study. C_{ext} refers to the nominal external concentration ($\mu mol\ L^{-}1$). V_{expo} is the volume of the exposure solution in ml. N_{ZFE} is the number of zebrafish embryos used in a single experimental replicate. N_{Trt} is the number of treatments applied for the given exposure concentration.N_{Obs} refers to the cumulative number of observations for all replicates and observation times.

Endpoint	ID_{Exp}	Year	N_{ZFE}	Substance	C_{ext}	V_{expo}	N_{Trt}	N_{Obs}
C_{int}	21	2016	9	diclofenac	5.0	18	2	34
C_{int}	21	2016	9	diclofenac	6.6	18	3	35
C_{int}	22	2016	9	diclofenac	6.6	18	6	65
C_{int}	22	2016	9	diclofenac	7.2	18	6	70
C_{int}	25	2017	9	diclofenac	6.6	18	2	44
C_{int}	32	2017	9	diclofenac	6.6	18	2	33
C_{int}	28	2017	9	diclofenac	6.6	18	6	83
C_{int}	29	2018	9	diclofenac	7.2	18	4	46
C_{int}	12	2020	9	diclofenac	7.4	18	1	28
C_{int}	33	2020	20	diclofenac	7.4	18	1	27
C_{int}	3	2021	20	diclofenac	7.4	6	1	33
C_{int}	19	2015	2	diuron	20.5	18	3	43
C_{int}	20	2016	9	diuron	20.0	18	3	30
C_{int}	20	2016	18	diuron	20.0	18	2	40
C_{int}	30	2018	8	diuron	20.0	18	1	2
C_{int}	30	2018	9	diuron	20.0	18	3	19
C_{int}	14	2021	20	diuron	28.1	18	1	27

Continued on next page

Table S1. Included experiment in the present study. C_{ext} refers to the nominal external concentration ($\mu mol\ L^{-}1$). V_{expo} is the volume of the exposure solution in ml. N_{ZFE} is the number of zebrafish embryos used in a single experimental replicate. N_{Trt} is the number of treatments applied for the given exposure concentration. N_{Obs} refers to the cumulative number of observations for all replicates and observation times.

Endpoint	ID_{Exp}	Year	N_{ZFE}	Substance	C_{ext}	V_{expo}	N_{Trt}	N_{Obs}
C_{int}	23	2017	9	naproxen	135.0	18	3	42
C_{int}	23	2017	9	naproxen	309.0	18	3	42
C_{int}	24	2017	9	naproxen	135.0	18	2	25
C_{int}	24	2017	9	naproxen	309.0	18	2	26
C_{int}	26	2017	9	naproxen	135.0	18	4	38
C_{int}	26	2017	9	naproxen	309.0	18	4	38
C_{int}	27	2017	9	naproxen	135.0	18	6	34
C_{int}	27	2017	9	naproxen	309.0	18	6	35
C_{int}	13	2020	20	naproxen	307.0	18	1	28
C_{int}	1	2021	12	naproxen	349.0	6	1	2
C_{int}	1	2021	20	naproxen	349.0	6	1	26
C_{int}	7	2022	12	naproxen	238.0	6	1	1
C_{int}	7	2022	20	naproxen	238.0	6	1	23
C_{int}	10	2022	20	naproxen	238.0	6	1	5
Nrf2	34	2016	10	diclofenac	5.1	18	2	8
Nrf2	34	2016	10	diclofenac	5.8	18	2	11
Nrf2	34	2016	10	diclofenac	6.5	18	2	9
Nrf2	34	2016	10	diclofenac	6.9	18	3	10
Nrf2	34	2016	10	diclofenac	7.4	18	2	12
Nrf2	35	2016	10	diclofenac	5.1	18	2	7
Nrf2	35	2016	10	diclofenac	5.8	18	2	7
Nrf2	35	2016	10	diclofenac	6.5	18	2	7
Nrf2	35	2016	10	diclofenac	6.9	18	2	6
Nrf2	35	2016	10	diclofenac	7.4	18	1	6
Nrf2	36	2016	10	diuron	2.3	18	2	7
Nrf2	36	2016	10	diuron	5.2	18	2	9
Nrf2	36	2016	10	diuron	11.7	18	2	9
Nrf2	36	2016	10	diuron	18.1	18	2	8
Nrf2	36	2016	10	diuron	29.4	18	2	12
Nrf2	36	2017	10	naproxen	135.0	18	2	8
Nrf2	36	2017	10	naproxen	178.0	18	1	6
Nrf2	36	2017	10	naproxen	234.0	18	2	8
Nrf2	36	2017	10	naproxen	269.0	18	2	8
Nrf2	36	2017	10	naproxen	309.0	18	2	11
phenotpye	40	2016	9	diclofenac	3.7	6	1	3
phenotpye	40	2016	9	diclofenac	4.6	6	1	3
phenotpye	40	2016	9	diclofenac	5.7	6	1	3
phenotpye	40	2016	9	diclofenac	7.2	6	1	3
phenotpye	40	2016	9	diclofenac	8.9	6	1	3
phenotpye	40	2016	9	diclofenac	11.2	6	1	3
phenotpye	40	2016	9	diclofenac	14.0	6	1	3
phenotpye	40	2016	9	diclofenac	17.5	6	1	3
phenotpye	40	2016	9	diclofenac	21.8	6	1	3
phenotpye	40	2016	9	diclofenac	27.3	6	1	3

Continued on next page

Table S1. Included experiment in the present study. C_{ext} refers to the nominal external concentration ($\mu mol\ L^{-}1$). V_{expo} is the volume of the exposure solution in ml. N_{ZFE} is the number of zebrafish embryos used in a single experimental replicate. N_{Trt} is the number of treatments applied for the given exposure concentration. N_{Obs} refers to the cumulative number of observations for all replicates and observation times.

Endpoint	ID_{Exp}	Year	N_{ZFE}	Substance	C_{ext}	V_{expo}	N_{Trt}	N_{Obs}
phenotpye	40	2016	9	diclofenac	34.1	6	1	3
phenotpye	40	2016	9	diclofenac	42.6	6	1	3
phenotpye	40	2016	9	diclofenac	53.3	6	1	3
phenotpye	41	2016	9	diclofenac	3.2	6	1	4
phenotpye	41	2016	9	diclofenac	3.8	6	1	4
phenotpye	41	2016	9	diclofenac	4.5	6	1	4
phenotpye	41	2016	9	diclofenac	5.4	6	1	4
phenotpye	41	2016	9	diclofenac	6.5	6	1	4
phenotpye	41	2016	9	diclofenac	7.8	6	1	4
phenotpye	41	2016	9	diclofenac	9.4	6	1	4
phenotpye	41	2016	18	diclofenac	11.3	6	1	4
phenotpye	41	2016	9	diclofenac	13.5	6	1	4
phenotpye	41	2016	9	diclofenac	16.2	6	1	4
phenotpye	41	2016	9	diclofenac	19.5	6	1	4
phenotpye	41	2016	9	diclofenac	23.4	6	1	4
phenotpye	41	2016	9	diclofenac	28.1	6	1	4
phenotpye	43	2015	9	diuron	5.3	6	1	2
phenotpye	43	2015	9	diuron	6.4	6	1	2
phenotpye	43	2015	9	diuron	7.8	6	1	2
phenotpye	43	2015	9	diuron	9.3	6	1	2
phenotpye	43	2015	9	diuron	11.2	6	1	2
phenotpye	43	2015	9	diuron	13.5	6	1	2
phenotpye	43	2015	9	diuron	15.7	6	1	2
phenotpye	43	2015	9	diuron	19.4	6	1	2
phenotpye	43	2015	9	diuron	23.3	6	1	2
phenotpye	43	2015	9	diuron	27.9	6	1	2
phenotpye	43	2015	9	diuron	33.5	6	1	2
phenotpye	43	2015	9	diuron	40.2	6	1	2
phenotpye	43	2015	9	diuron	57.9	6	1	2
phenotpye	43	2015	9	diuron	69.5	6	1	2
phenotpye	43	2015	9	diuron	83.4	6	1	2
phenotpye	44	2015	9	diuron	2.1	6	1	2
phenotpye	44	2015	9	diuron	3.2	6	1	2
phenotpye	44	2015	9	diuron	4.8	6	1	2
phenotpye	44	2015	9	diuron	7.2	6	1	2
phenotpye	44	2015	9	diuron	8.5	6	1	2
phenotpye	44	2015	9	diuron	10.8	6	1	2
phenotpye	44	2015	9	diuron	12.8	6	1	2
phenotpye	44	2015	9	diuron	14.9	6	1	2
phenotpye	44	2015	9	diuron	16.1	6	1	2
phenotpye	44	2015	9	diuron	24.2	6	1	2
phenotpye	44	2015	9	diuron	36.3	6	1	2
phenotpye	44	2015	9	diuron	54.5	6	1	2
phenotpye	44	2015	9	diuron	81.7	6	1	2

Continued on next page

Table S1. Included experiment in the present study. C_{ext} refers to the nominal external concentration ($\mu mol\ L^{-}1$). V_{expo} is the volume of the exposure solution in ml. N_{ZFE} is the number of zebrafish embryos used in a single experimental replicate. N_{Trt} is the number of treatments applied for the given exposure concentration. N_{Obs} refers to the cumulative number of observations for all replicates and observation times.

Endpoint	ID_{Exp}	Year	N_{ZFE}	Substance	C_{ext}	V_{expo}	N_{Trt}	N_{Obs}
phenotpye	47	2016	18	naproxen	10.6	6	1	4
phenotpye	47	2016	18	naproxen	21.2	6	1	4
phenotpye	47	2016	18	naproxen	42.3	6	1	4
phenotpye	47	2016	18	naproxen	84.7	6	1	4
phenotpye	47	2016	18	naproxen	169.0	6	1	4
phenotpye	47	2016	18	naproxen	339.0	6	1	4
phenotpye	47	2016	18	naproxen	677.0	6	1	4
phenotpye	47	2016	18	naproxen	1350.0	6	1	4
phenotpye	48	2016	18	naproxen	282.0	6	1	3
phenotpye	48	2016	18	naproxen	338.0	6	1	3
phenotpye	48	2016	18	naproxen	405.0	6	1	3
phenotpye	48	2016	18	naproxen	487.0	6	1	3
phenotpye	48	2016	18	naproxen	584.0	6	1	3
phenotpye	48	2016	18	naproxen	701.0	6	1	3
phenotpye	48	2016	18	naproxen	841.0	6	1	3
phenotpye	48	2016	18	naproxen	1010.0	6	1	3
phenotpye	48	2016	18	naproxen	1210.0	6	1	3
phenotpye	48	2016	18	naproxen	1450.0	6	1	3
phenotpye	49	2016	18	naproxen	137.0	6	1	3
phenotpye	49	2016	18	naproxen	165.0	6	1	3
phenotpye	49	2016	18	naproxen	198.0	6	1	3
phenotpye	49	2016	18	naproxen	237.0	6	1	3
phenotpye	49	2016	18	naproxen	285.0	6	1	3
phenotpye	49	2016	18	naproxen	342.0	6	1	3
phenotpye	49	2016	18	naproxen	410.0	6	1	3
phenotpye	49	2016	18	naproxen	492.0	6	1	3

S7 Parameter estimation

S7.1 Bayesian parameter inference

Working with temporally resolved exposure and 'omics data means integrating different datasets from numerous biological experiments into the modeling and optimization/inference process. This is associated with considerable experimental noise, which complicates the process of obtaining accurate parameters that determine the model. Accounting for measurement error and biological stochasticity (e.g. in lethality or sublethal effects) in the data can help to infer the true effect of a chemical by separating noise from effect. Bayesian parameter inference approaches accommodate all these necessities and, further, report parameter distributions, reflecting the uncertainty in the true parameter and also provide estimates of the expected variation in experimental observations. These so called, posterior predictions, are excellent tools for gauging the predictive capacities of the model and on top of that provide an ideal validation tool for novel incoming data. At the heart of the Bayesian philosophy is a process that is called Bayesian updating. In order to explain this a short excurse into the Bayes rule is necessary.

$$Pr(\theta \mid Y) = \frac{Pr(\theta)\ Pr(Y \mid \theta)}{Pr(Y)} \qquad \text{(Eq. S14)}$$

$$Posterior \approx Prior \times Likelihood \qquad \text{(Eq. S15)}$$

Eq. S14 is also known as Bayes Theorem and it is used to calculate conditional probabilities. It reads as: The probability of a set of parameters θ, conditional on the observed data Y is equal to the joint probability of the parameters and the probability of the likelihood of the data given the parameters (and the model to relate parameters to data), divided by the probability of the observations Y. Because the calculation of the denominator $Pr(Y)$ of the equation is complicated, it is usually ignored due to its independence of the parameters (no θ is involved) and considered as a proportionality constant, which ensures that the resulting probability function integrates to 1. Thus, Eq. S15 contains the remaining components of Eq. S14 that actually bear relevant concepts for the understanding of uncertainty in statistics—*posterior*, *prior* and *likelihood.* The notation of the likelihood in Eq. S14 is a convention. One could also rewrite the likelihood as $Pr(Y \mid f(\theta))$ to more explicitly express that the likelihood of the data not only depends on the parameter values but also on the used mathematical model f, that describes how the parameters θ are transformed to predict the observed data Y. To be clear, in the scope of this work f is a TKTD model. For more information on the theory of bayesian inference, we refer to the excellent handbook *Statistical Rethinking* [5].

S7.2 Leveraging modern probabilistic programming languages (PPL) to solve the computational challenges of the 'omics integration into TKTD models

The difficulty of performing Bayesian parameter inference on an ODE model is that systems of ODEs need to be solved 10,000–100,000s of times in order to first converge on the typical set of parameters, and then sample frequently enough to build a valid approximation of the posterior parameter distribution. Such a procedure may take a long time, if the ODE system is solved in each iteration. Fortunately, modern probabilistic programming languages (PPL) provide the tools, to address such difficulties very efficiently, by exploiting auto differentiation with an *adjoint sensitivity* approach and by applying compilation to highly efficient symbolic languages. In this work, *numpyro* [6, 7] was used as a PPL (other highly recommendable choices are Stan, or pymc). Numpyro uses *JAX* [8] to compile ODE models and deliver solutions along with autodifferentiated gradients, with respect to the model parameters. By using JAX, the 202 ODE systems needed to integrate all datasets into one model could be evaluated very efficiently resulting in a model evaluation time of 40 ms for 1 iteration. Still a computational problem remains, because for using the state of the art MCMC method, NUTS [9], the likelihood function (and its gradients) need to be computed for each data point. In the given dataset, this means close to 1000 gradient evaluations with respect to all model parameters per leapfrog step (the number of leapfrosteps

varied between 1–1023 per iteration). This easily scales to dimensions where gradient based MCMC approaches, like NUTS have difficulties, especially when the ODE model and therefore the likelihood function and its gradients, becomes more complex. For simple problem like the 4-parameter GUTS model k_d, k_k, h_b, z, solving the problem with a NUTS approach is feasible (walltime ≈ 30 minutes), but with more complex models with higher number of parameters, NUTS approaches quickly becomes infeasible (walltime > 48 h). In these situation, posteriors were approximated with stochastic variational inference (SVI) [10], which estimates posterior distributions, based on finding a parametric distribution that approximates the true, unknown posterior distribution. While these methods, are constrained to deliver parametric posteriors, they were in good agreement with the posteriors produced by the NUTS algorithm. In order to address the prerequisites of highly fragmented and complex datasets for bayesian parameter inference, `pymob` (`https://github.com/flo-schu/pymob`) is being developed as a modeling framework, which allows the user to switch between inference frameworks (e.g. interactive, maximum-likelihood, approximate-bayes, fully-bayesian), while maintaining a consistent deterministic and stochastic model formulation. In essence, to minimize the frustrating dimensional overhead when working with complex datasets and seamlessly switch between different tools, which may work better or worse with different demands of the data and model.

In order to assess parameter uncertainty and identify potential identifiability issues, 100 markov chains (NUTS) or 100 SVI approximations, were started with initial parameters drawn from a uniform interval from -1 to 1, which were subsequently transformed to the scales priors of the parameter distributions. This effectively detects any local minimima with likelihoods very close to the global optimum and thus indicate the presence of parameter identifiability issues. The exact algorithm is described in Section S7.3.

S7.3 Parameter analysis algorithm

The parameter uncertainty analysis algorithm follows these steps:

1. Start 100 parameter estimations with stochastic variational inference (SVI) with a multivariate normal distribution that maps (is transformed) onto the lognormal distributions containing the prior information. The starting values are drawn from a uniform distribution between [-1, 1], which are subsequently transformed to the prior distribution, resulting in a sampling interval of $\left[e^{-1 \cdot s + \mu_\theta},\ e^{1 \cdot s + \mu_\theta}\right]$, where μ_θ is the mode of the prior distribution and s is the standard deviation of the prior distribution on the logarithmic scale (here $s = 2$ for all prior parameter distributions of the deterministic model). This will cover the majority of the prior density. The interval is not selected larger, because already this range contains parameter estimates that impose severe difficulties for the solver. SVI is parameterized with a learning rate of 0.001 and 50,000 iterations. Usually convergence happens within the first few 1000 iterations. The algorithm skips updates of the estimator containing infeasible values (infinity, nan-values; these occur, when the solver reaches the maximum number of steps because of occasional extreme parameter combinations).

2. After 4 hours, all running estimations are terminated, because in all probability they were stuck on some very slow local minimum, where evaluations of the deterministic solver takes a long time. Samples of the 100 estimators are combined into one large posterior and saved. This leads to a multidimensional posterior with 100 independently trained estimators, so called (Markov) chains if MCMC is used, and 2000 parameter samples per estimator per model parameter. This is typical for Bayesian posterior samples.

3. Following this, the posterior samples are clustered, by comparing whether the parameter means are within 1 standard deviation of any other, independently sampled parameter distribution of the remaining 99 estimators.

4. Next, clusters are filtered so that only those clusters are retained that have a log-likelihood less negative than 1.1 times the least negative log likelihood. This ensures that only these clusters remain that are close to the optimum found in the 100 estimations (Note that this does not preclude the possibility that a global optimum is never found, but it is at least very unlikely).

5. Calculate Bayesian Information Criteria (BIC) write a table (Tex) of the parameters and their standard deviations. Plot log-histograms of the parameters, plot joint distributions of pairwise parameters to visually inspect clusters, plot posterior predictions of the different substances. These estimates are used solely for model improvement and understanding the different stable modes in the case of parameter-identifiability issues.

6. Finally, the same procedure as described in (4 and 5) is conducted for all estimates with a likelihood deviation of 1.025 (2.5%) below the estimate with the highest likelihood. In plain words, we are left with N fully independent parameter estimates (including their full uncertainty) that have converged to or close to the global optimum.

This estimates within 2.5% variation of the best estimtate are used for reporting in the study. It should reflect the uncertainty into the model parameters very well. In case not many chains are in this estimate, it indicates that the number of iterations were too few, or the learning rate too low, or the prior distributions too wide. Ideally estimation should be repeated until a reasonable number of independent estimates (> 5) have converged on the same maximal likelihood. Of course this is still no guarantee that the global best estimate has been found, but it is a good indication.

S8 GUTS-RNA-pulse model

S8.1 Model description of the GUTS-RNA-pulse model (substance specific and substance independent)

$$\frac{dC_i}{dt} = k_i\ C_e - k_m\ C_i\ P \qquad \text{(Eq. S16)}$$

$$\frac{dR}{dt} = r_{rt}\ \text{activation}(C_i,\ C_{i,max},\ z_{ci},\ v_{rt}) - k_{rd}\ (R - R_0) \qquad \text{(Eq. S17)}$$

$$\frac{dP}{dt} = k_p\ ((R - R_0) - P) \qquad \text{(Eq. S18)}$$

$$h(t) = k_k\ max(0, R(t) - z) + h_b \qquad \text{(Eq. S19)}$$

$$S(t) = e^{-\int_0^t h(t)dt} \qquad \text{(Eq. S20)}$$

with

$$activation(C_i,\ C_{i,max},\ z_{ci},\ v_{rt}) = 0.5 + \frac{1}{\pi}\ arctan(v_{rt}\ (\frac{C_i}{C_{i,max}} - z_{ci})) \qquad \text{(Eq. S21)}$$

Table S2. TKTD state variables and parameters used in the GUTS-RNA-pulse model. The column "Assumed substance independence" indicates whether a parameter is supposed to be shared for multiple substances.

Symbol	Definition	Unit	Assumed substance independence
Model state variables			
C_e	Environmental concentration in the aqueous medium	$\mu mol\ L^{-1}$	
C_i	Internal concentration of the homogenized ZFE	$\mu mol\ L^{-1}$	
R	Relative differential RNA transcription in the ZFE	fc [c]	
R_0	Relative differential initial RNA transcription in the ZFE	fc [c]	
P^*	Scaled protein concentration in the ZFE	fc [c]	
h	Instantaneous hazard rate at time t	h^{-1}	
S	Survival probability of a ZFE at time t	–	
Model parameters			
k_i	Uptake rate constant of the chemical into the internal compartment of the ZFE	h^{-1}	no
k_m	Scaled metabolization rate constant from the internal compartment of the ZFE	h^{-1}	no
z_{ci}	Scaled internal concentration threshold for the activation of *nrf2* expression	– [d]	no
v_{rt}	Scaled responsiveness of the *nrf2* activation (slope of the activation function)	– [d]	yes/no [a]
r_{rt}	Constant *nrf2* expression rate after activation [b]	fc [c]	yes
k_{rd}	Nrf2 decay rate constant	h^{-1}	yes
k_p	Dominant rate constant of synthesis and decay of metabolizing proteins	h^{-1}	yes
z	Effect *nrf2*-threshold of the hazard function [b]	fc [c]	yes
k_k	killing rate constant for *nrf2* [b]	$\text{fc}^{-1}\ h^{-1}$ [c]	yes
h_b	background hazard rate	h^{-1}	yes
σ_{cint}	Standard deviation of the lognormal distribution of the internal concentration	–	yes
σ_{nrf2}	Standard deviation of the lognormal distribution of the *nrf2* expression [b]	–	yes

[a] In an unscaled version of the activation function, v_{rt} is not considered substance independent, due to an inverse relationship between v_{rt} and $C_{i,max}$

[b] relative to the *nrf2* concentration in untreated ZFE (fold-change)

[c] fc: fold change $\frac{\mu mol\ nrf2\text{-treatment}\ L^{-1}}{\mu mol\ nrf2\text{-control}\ L^{-1}}$

[d] scaled internal concentrations: $\frac{\mu mol\ C_i(t)\ L^{-1}}{\mu mol\ C_{i,max}\ L^{-1}}$

S8.2 Model fits for GUTS-RNA-pulse

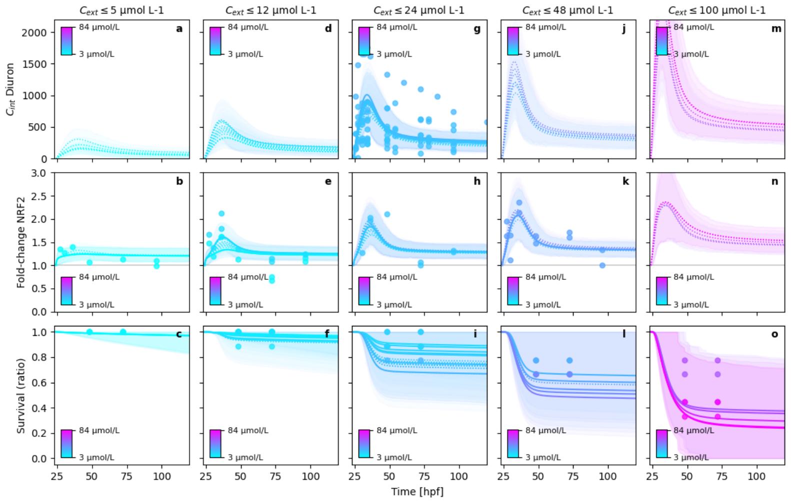

Figure S3. Posterior estimates and 95%-BCIs of the GUTS-RNA-pulse model for diuron

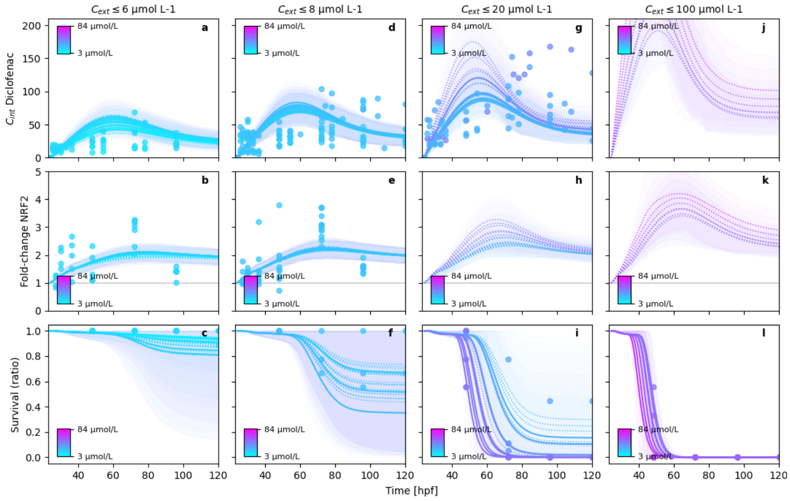

Figure S4. Posterior estimates and 95%-BCIs of the GUTS-RNA-pulse model for diclofenac

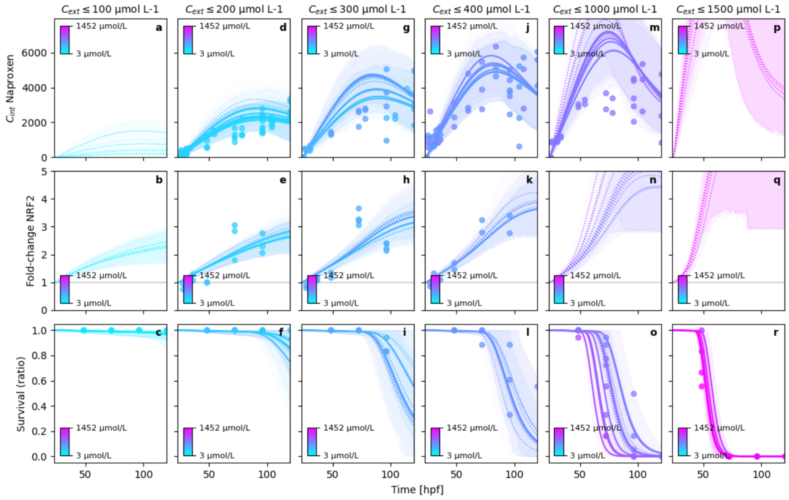

Figure S5. Posterior estimates and 95%-BCIs of the GUTS-RNA-pulse model for naproxen

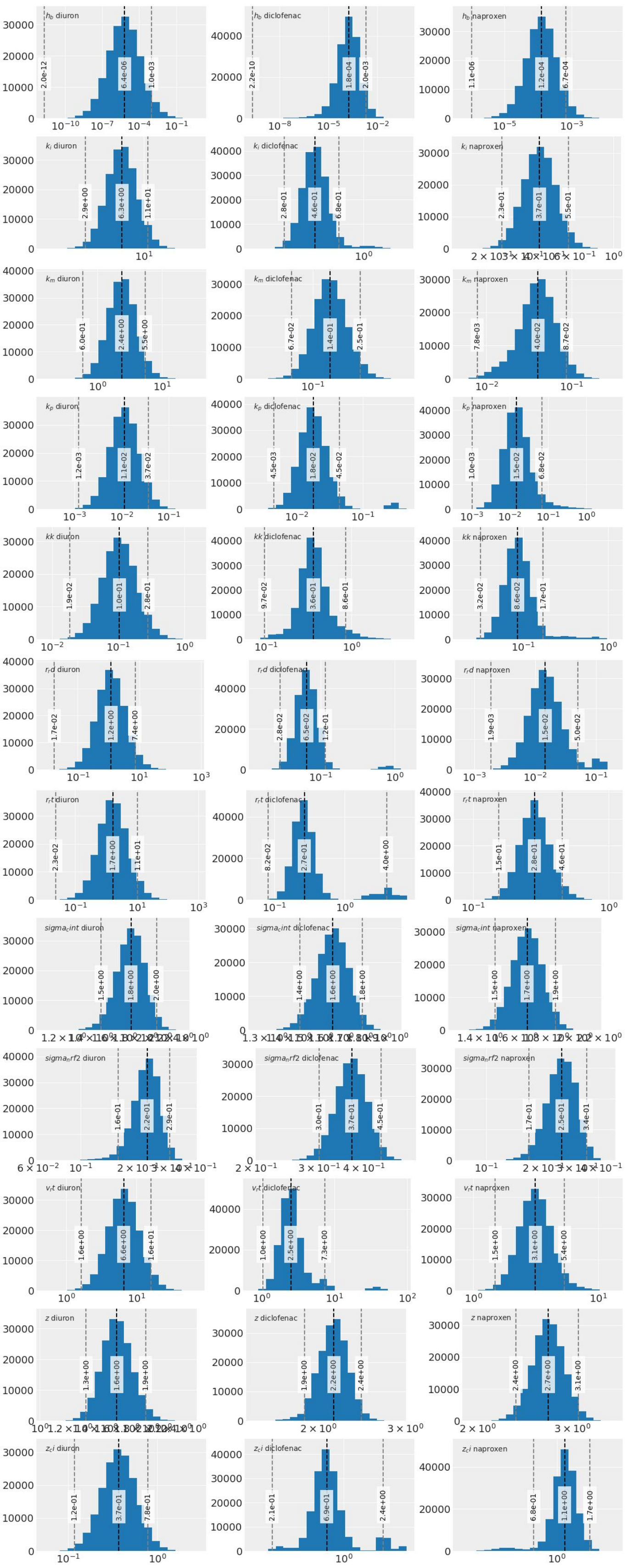

Figure S6. Parameter estimates of the GUTS-RNA-pulse model with substance specific parameters.

Table S3. Parameter estimates and posterior highest densitiy intervals (HDI) of the substance specific GUTS-RNA-pulse model. The HDI contains 94% of the probable parameter values given the data. fc = fold-change (*nrf2*).

Parameter	Diuron			Diclofenac			Naproxen		
	mean	hdi 3%	hdi 97%	mean	hdi 3%	hdi 97%	mean	hdi 3%	hdi 97%
k_i (h^{-1})	6.62	3.06	10.69	0.48	0.29	0.66	0.38	0.24	0.54
k_m (h^{-1})	2.71	0.69	5.30	0.15	0.07	0.24	0.04	0.01	0.08
z_{ci} ($-$)	0.41	0.12	0.75	0.84	0.21	2.28	1.14	0.72	1.70
v_{rt} ($-$)	7.60	1.77	15.30	3.63	1.03	6.55	3.32	1.53	5.19
r_{rt} (fc)	3.28	0.04	9.64	0.65	0.09	3.63	0.30	0.15	0.44
k_{rd} (h^{-1})	2.28	0.02	6.60	0.08	0.03	0.11	0.02	0.00	0.04
k_p (h^{-1})	0.01	0.00	0.03	0.03	0.00	0.04	0.03	0.00	0.06
z (fc)	1.61	1.34	1.90	2.15	1.89	2.43	2.71	2.36	3.06
k_k (fc^{-1} h^{-1})	0.12	0.02	0.27	0.41	0.09	0.79	0.10	0.03	0.16
h_b (h^{-1})	0.00	0.00	0.00	0.00	0.00	0.00	0.00	0.00	0.00
σ_{cint} ($-$)	1.76	1.49	2.01	1.61	1.46	1.76	1.67	1.49	1.86
σ_{nrf2} ($-$)	0.22	0.16	0.29	0.37	0.30	0.44	0.25	0.17	0.33

S8.3 Model fits for GUTS-RNA-pulse model with parameter sharing for the RNA and protein modules

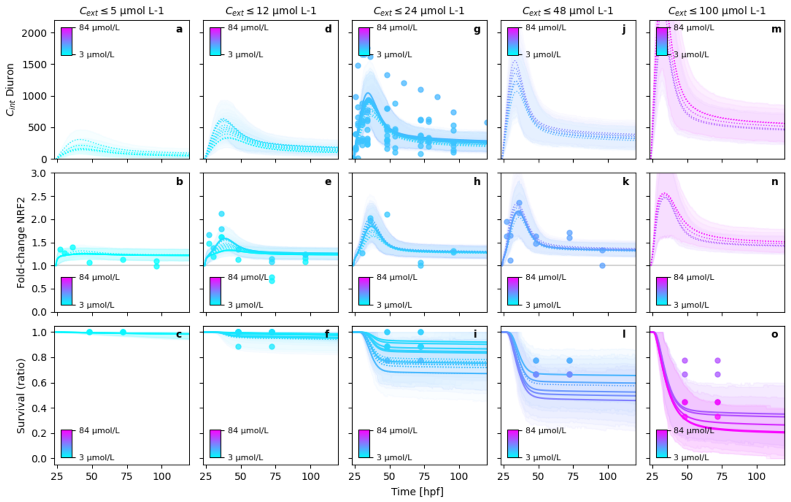

Figure S7. Posterior estimates and 95%-BCIs of diuron for the parameter sharing GUTS-RNA-pulse model.

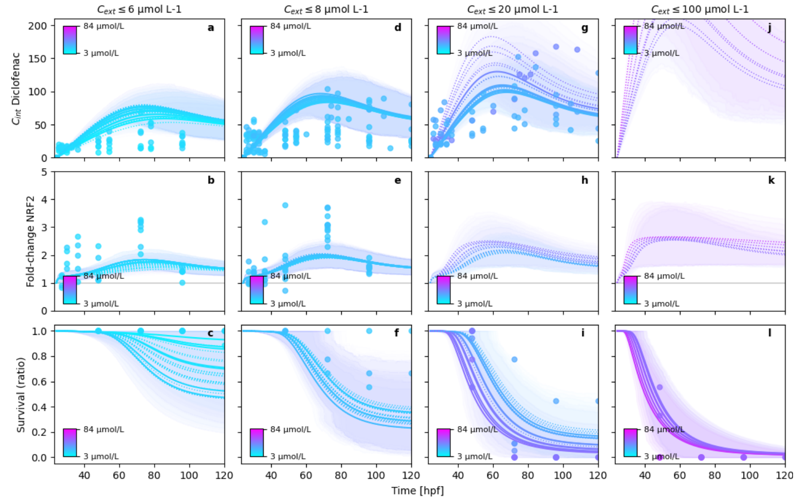

Figure S8. Posterior estimates and 95%-BCIs of diclofenac for the parameter sharing GUTS-RNA-pulse model.

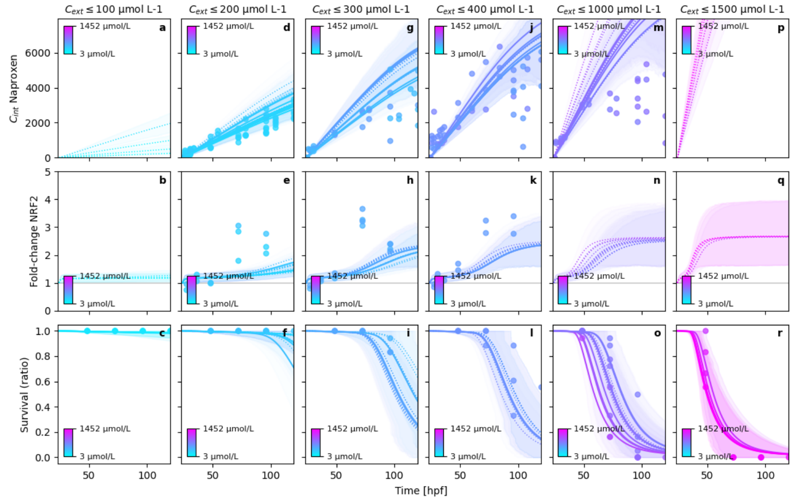

Figure S9. Posterior estimates and 95%-BCIs of naproxen for the parameter sharing GUTS-RNA-pulse model.

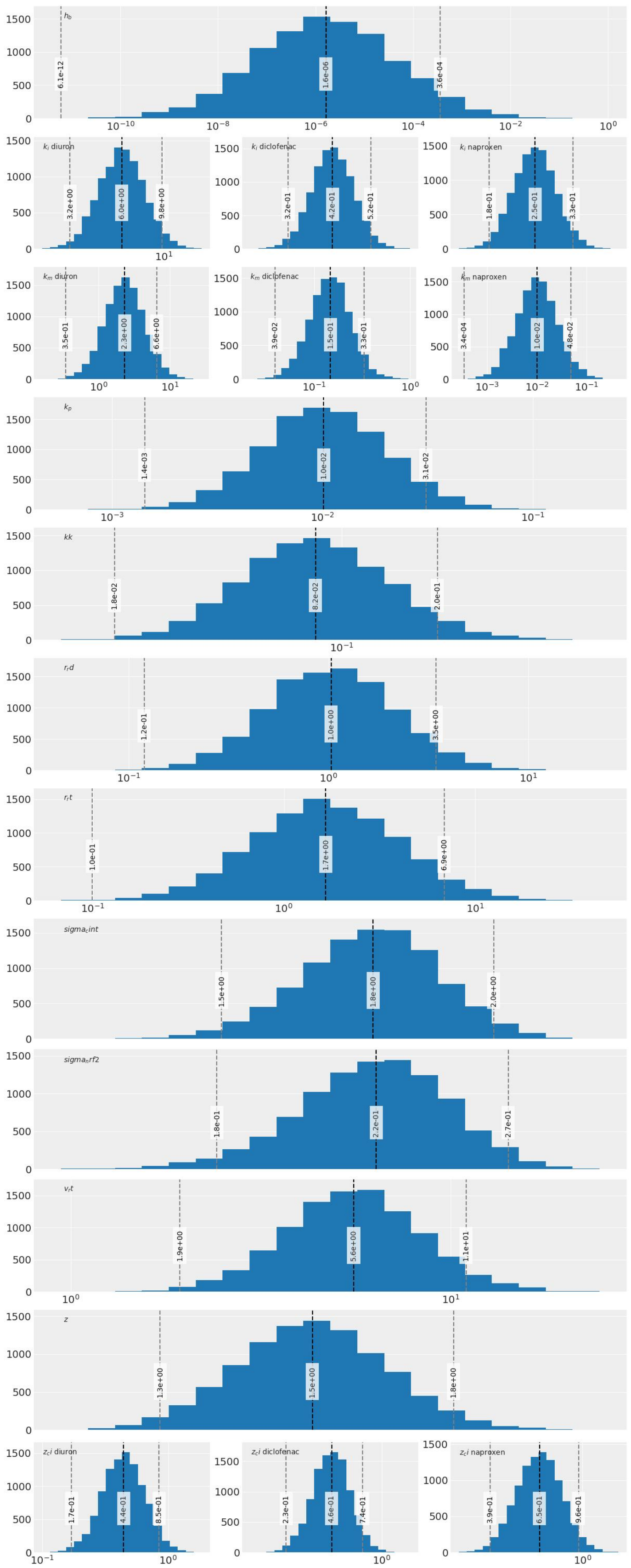

Figure S10. Parameter estimates of the GUTS-RNA-pulse model with substance-independent parameters for the RNA and protein dynamics.

Table S4. Parameter estimates and posterior highest densitiy intervals (HDI) of the GUTS-RNA-pulse model with a substance independent RNA protein module. Parameters which share information between substances are given in the form (mean (3% hdi–97% hdi)). Parameter sharing reduces the number of parameters for all 3 substances from 30 to 18. fc = fold-change (*nrf2*).

Parameter	Diuron			Diclofenac			Naproxen		
	mean	hdi 3%	hdi 97%	mean	hdi 3%	hdi 97%	mean	hdi 3%	hdi 97%
k_i (h^{-1})	6.21	3.29	9.67	0.42	0.33	0.52	0.25	0.19	0.33
k_m (h^{-1})	2.83	0.41	6.26	0.17	0.04	0.32	0.02	0.00	0.04
z_{ci} ($-$)	0.48	0.17	0.82	0.47	0.24	0.73	0.66	0.39	0.94
v_{rt} ($-$)				6.02 (2.13–10.76)					
r_{rt} (fc)				2.42 (0.08–6.34)					
k_{rd} (h^{-1})				1.35 (0.13–3.27)					
k_p (h^{-1})				0.01 (0.00–0.03)					
z (fc)				1.55 (1.30–1.80)					
k_k (fc^{-1} h^{-1})				0.10 (0.02–0.20)					
h_b (h^{-1})				0.00 (0.00–0.00)					
σ_{cint} ($-$)				1.76 (1.52–1.99)					
σ_{nrf2} ($-$)				0.22 (0.18–0.27)					

S9 GUTS-RNA model

S9.1 Model description of the GUTS-RNA model

$$\frac{dC_i}{dt} = k_i\ C_e - k_e\ C_i \quad \text{(Eq. S22)}$$

$$\frac{dR}{dt} = k_a\ C_i - k_r\ R \quad \text{(Eq. S23)}$$

$$h(t) = k_k\ max(0, R(t) - z) + h_b \quad \text{(Eq. S24)}$$

$$S(t) = e^{-\int_0^t h(t)dt} \quad \text{(Eq. S25)}$$

Table S5. TKTD Parameters used in the GUTS-RNA model.

Parameter	Definition	Unit
Model state variables		
C_e	Environmental concentration in the aqueous medium	$\mu mol\ L^{-1}$
C_i	Internal concentration of the homogenized ZFE	$\mu mol\ L^{-1}$
R	Relative differential RNA transcription in the ZFE	fc [c]
h	Instantaneous hazard rate at time t	h^{-1}
S	Survival probability of a ZFE at time t	–
Model parameters		
k_i	Uptake rate constant of the chemical into the internal compartment of the ZFE	h^{-1}
k_e	Elimination rate constant from the internal compartment of the ZFE	h^{-1}
k_a	Damage accrual rate constant	fc $L\ \mu mol^{-1}\ h^{-1}$ [b]
k_r	Damage repair rate constant	h^{-1}
z	Effect *nrf2*-threshold of the hazard function [a]	fc [b]
k_k	killing rate constant for *nrf2* [a]	fc^{-1} h^{-1} [b]
h_b	background hazard rate constant	h^{-1}
σ_{cint}	Standard deviation of the lognormal distribution of the internal concentration	–
σ_{nrf2}	Standard deviation of the lognormal distribution of the *nrf2* expression [a]	–

[a] relative to the *nrf2* concentration in untreated ZFE (fold-change)
[b] fold change: $\frac{\mu mol\ nrf2\text{-treatment}\ L^{-1}}{\mu mol\ nrf2\text{-control}\ L^{-1}}$

S9.2 Model fits for GUTS-RNA

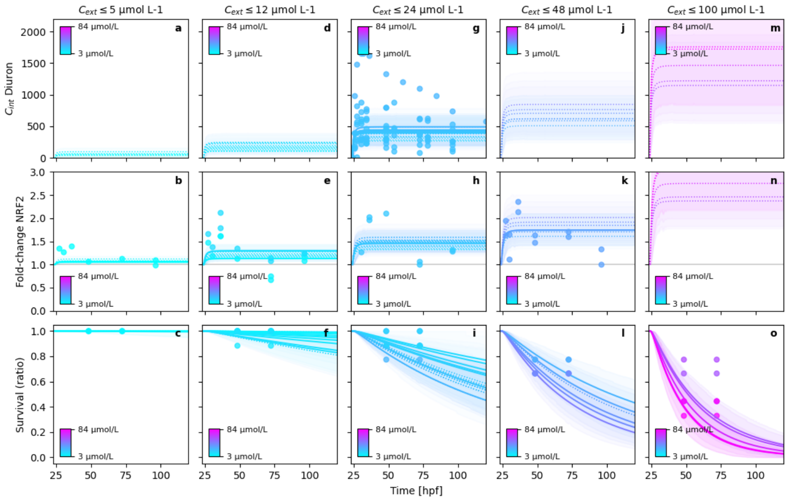

Figure S11. Posterior estimates and 95%-BCIs of diuron for GUTS-RNA model.

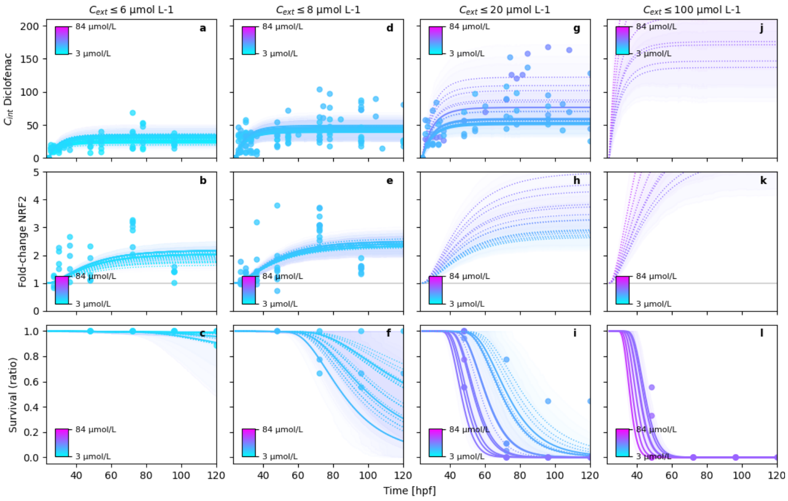

Figure S12. Posterior estimates and 95%-BCIs of diclofenac for GUTS-RNA model.

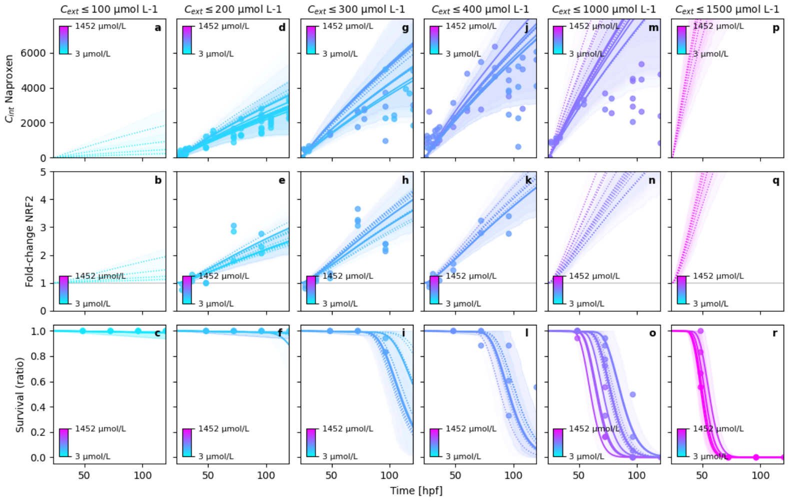

Figure S13. Posterior estimates and 95%-BCIs of naproxen for GUTS-RNA model.

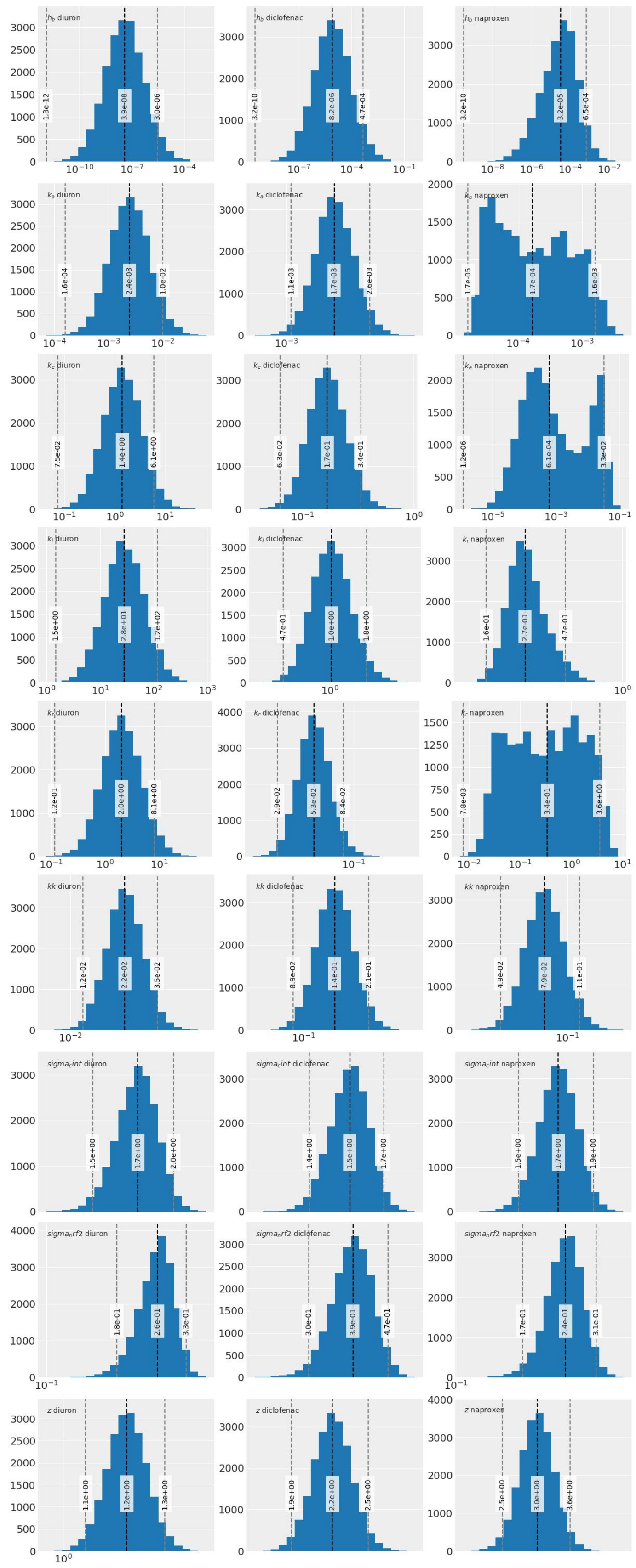

Figure S14. Parameter estimates of the GUTS-RNA model.

Table S6. Parameter estimates and posterior highest density intervals (HDI) of the GUTS-RNA model. The HDI contains 94% of the probable parameter values given the data. fc = fold-change (*nrf2*).

Parameters	Diuron			Diclofenac			Naproxen		
	mean	hdi 3%	hdi 97%	mean	hdi 3%	hdi 97%	mean	hdi 3%	hdi 97%
k_i (h^{-1})	41.74	1.48	110.75	1.08	0.49	1.74	0.29	0.16	0.45
k_e (h^{-1})	2.11	0.08	5.61	0.18	0.07	0.32	0.01	0.00	0.03
k_a ($\frac{\text{fc } L}{\mu mol\ h}$)	0.00	0.00	0.01	0.00	0.00	0.00	0.00	0.00	0.00
k_r (h^{-1})	2.87	0.10	7.47	0.05	0.03	0.08	0.89	0.01	3.33
z (fc)	1.19	1.07	1.31	2.19	1.89	2.47	3.03	2.55	3.53
k_k (fc^{-1} h^{-1})	0.02	0.01	0.03	0.14	0.09	0.20	0.08	0.05	0.11
h_b (h^{-1})	0.00	0.00	0.00	0.00	0.00	0.00	0.00	0.00	0.00
σ_{cint} ($-$)	1.74	1.48	1.99	1.53	1.38	1.68	1.72	1.53	1.91
σ_{nrf2} ($-$)	0.26	0.19	0.33	0.39	0.31	0.47	0.24	0.18	0.31

S10 GUTS-scaled-damage model

S10.1 Model description of the GUTS-scaled-damage model

$$\frac{dC_i}{dt} = k_i\ C_e - k_e\ C_i \quad \text{(Eq. S26)}$$

$$\frac{dD}{dt} = k_d \cdot (C_i - D) \quad \text{(Eq. S27)}$$

$$h(t) = k_k\ max(0, D(t) - z) + h_b \quad \text{(Eq. S28)}$$

$$S(t) = e^{-\int_0^t h(t)dt} \quad \text{(Eq. S29)}$$

Table S7. TKTD Parameters used in the GUTS-scaled-damage model.

Parameter	Definition	Unit
Model state variables		
C_e	Environmental concentration in the aqueous medium	$\mu mol\ L^{-1}$
C_i	Internal concentration of the homogenized ZFE	$\mu mol\ L^{-1}$
D	Scaled damage	$\mu mol\ L^{-1}$
h	Instantaneous hazard rate at time t	h^{-1}
S	Survival probability of a ZFE at time t	–
Model parameters		
k_i	Uptake rate constant of the chemical into the internal compartment of the ZFE	h^{-1}
k_e	Elimination rate constant from the internal compartment of the ZFE	h^{-1}
k_d	Dominant rate constant of damage dynamics	h^{-1}
z	Effect damage-threshold of the hazard function	$\mu mol\ L^{-1}$
k_k	killing rate constant	$L\ \mu mol^{-1}\ h^{-1}$
h_b	background hazard rate constant	h^{-1}
σ_{cint}	Standard deviation of the lognormal distribution of the internal concentration	–

S10.2 Model fits for GUTS-scaled-damage

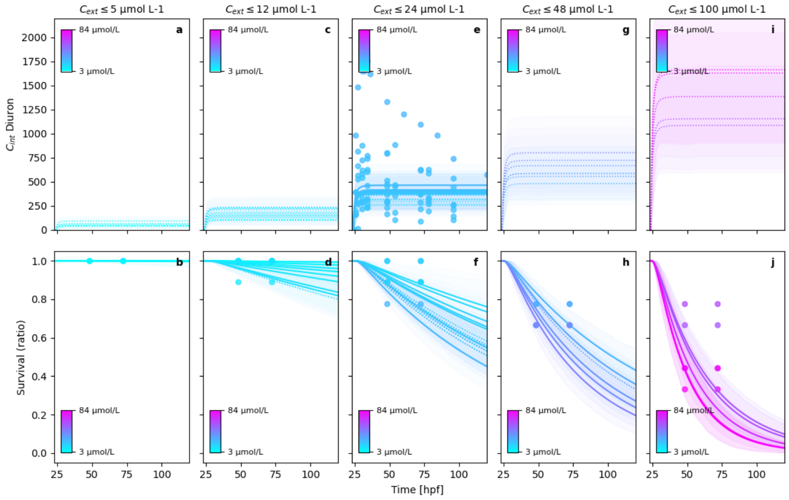

Figure S15. Posterior estimates and 95%-BCIs of diuron for GUTS-scaled damage model.

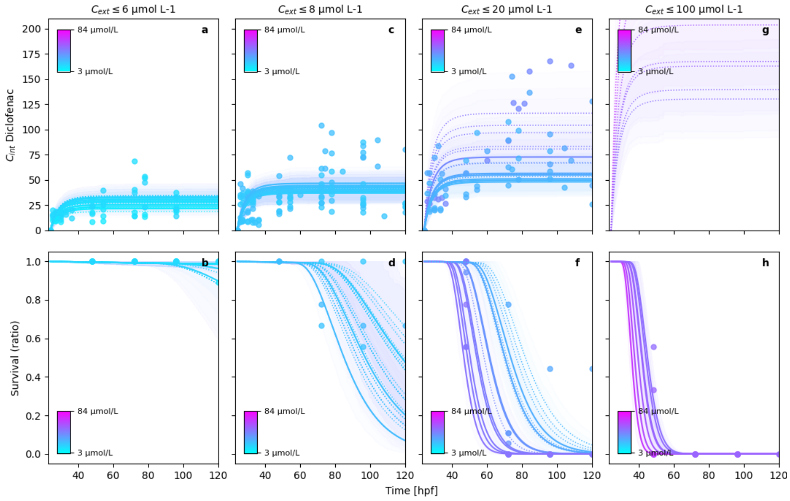

Figure S16. Posterior estimates and 95%-BCIs of diclofenac for GUTS-scaled damage model.

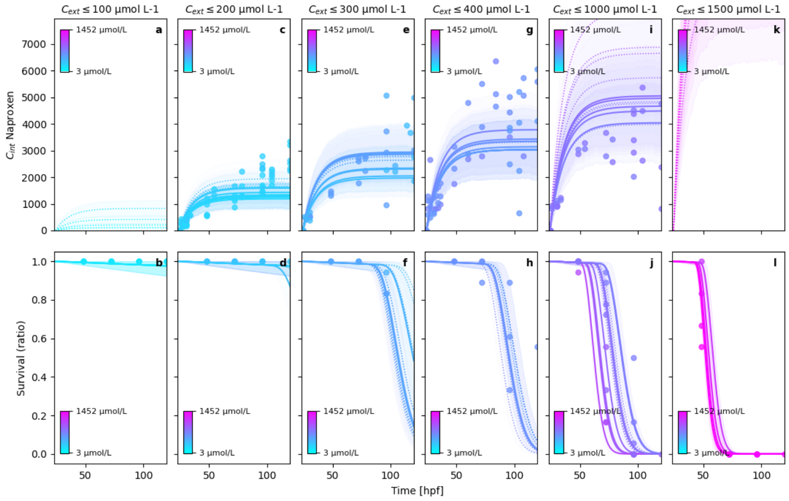

Figure S17. Posterior estimates and 95%-BCIs of naproxen for GUTS-scaled damage model.

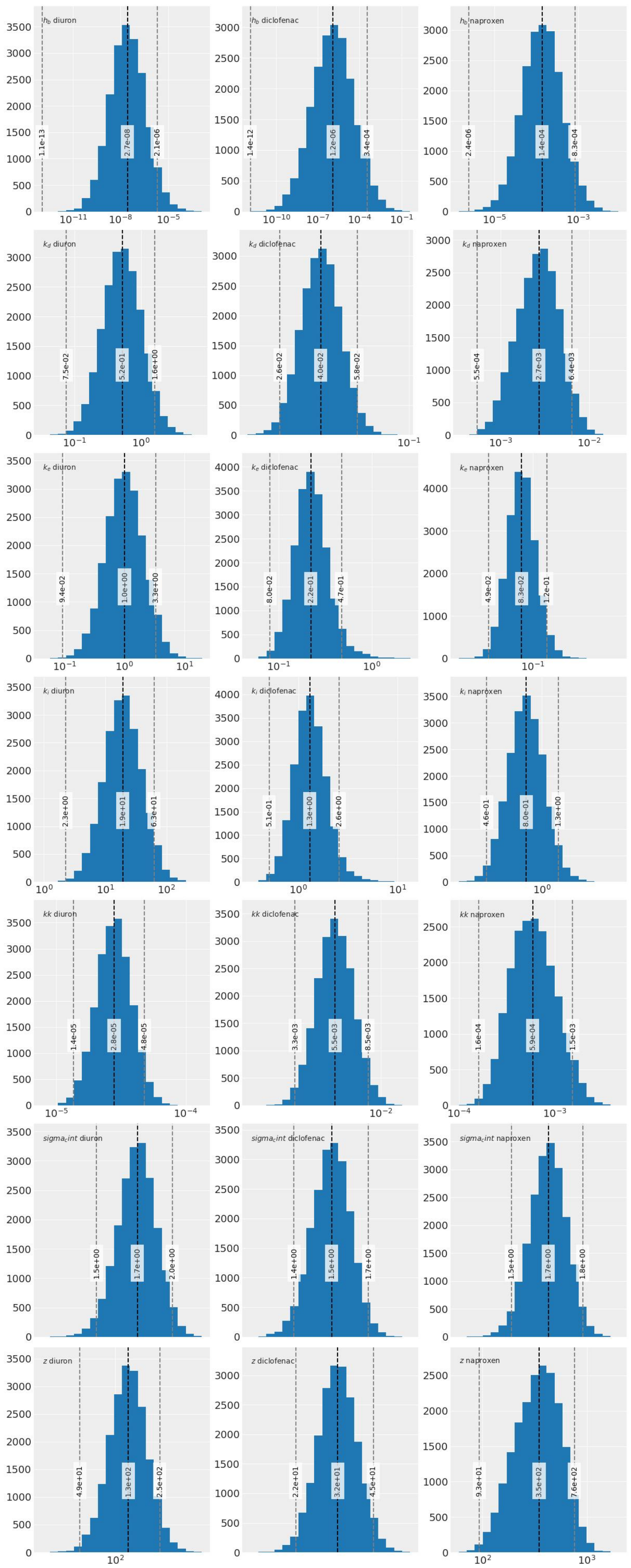

Figure S18. Parameter estimates of the GUTS-scaled-damage model with substance specific parameters.

Table S8. Parameter estimates and posterior highest densitiy intervals (HDI) of the GUTS-scaled-damage model. The HDI contains 94% of the probable parameter values given the data.

Parameters	Diuron			Diclofenac			Naproxen		
	mean	hdi 3%	hdi 97%	mean	hdi 3%	hdi 97%	mean	hdi 3%	hdi 97%
k_i (h^{-1})	25.01	2.94	59.77	1.44	0.53	2.49	0.83	0.46	1.23
k_e (h^{-1})	1.30	0.11	3.13	0.25	0.08	0.45	0.09	0.05	0.12
k_d (h^{-1})	0.66	0.08	1.52	0.04	0.03	0.06	0.00	0.00	0.01
z $(\mu mol\ L^{-1})$	139.63	52.23	241.07	32.67	21.99	44.61	384.24	90.52	726.16
k_k $(\frac{L}{\mu mol\ h})$	0.00	0.00	0.00	0.01	0.00	0.01	0.00	0.00	0.00
h_b (h^{-1})	0.00	0.00	0.00	0.00	0.00	0.00	0.00	0.00	0.00
σ_{cint} $(-)$	1.73	1.50	1.97	1.52	1.39	1.64	1.65	1.49	1.81

S11 GUTS-reduced model

S11.1 Model description of the GUTS-reduced model

$$\frac{dD}{dt} = k_d \cdot (C_e - D) \quad \text{(Eq. S30)}$$

$$h(t) = k_k \; max(0, D(t) - z) + h_b \quad \text{(Eq. S31)}$$

$$S(t) = e^{-\int_0^t h(t)dt} \quad \text{(Eq. S32)}$$

Table S9. TKTD state variables and parameters used in the GUTS-reduced model.

Parameter	Definition	Unit
Model state variables		
C_e	Environmental concentration in the aqueous medium	$\mu mol\ L^{-1}$
D	Scaled damage	$\mu mol\ L^{-1}$
h	Instantaneous hazard rate at time t	h^{-1}
S	Survival probability of a ZFE at time t	–
Model parameters		
k_d	Dominant rate constant of damage dynamics	h^{-1}
z	Effect damage-threshold of the hazard function	$\mu mol\ L^{-1}$
k_k	killing rate constant	$L\ \mu mol^{-1}\ h^{-1}$
h_b	background hazard rate constant	h^{-1}
σ_{cint}	Standard deviation of the lognormal distribution of the internal concentration	–

S11.2 Model fits for GUTS-reduced

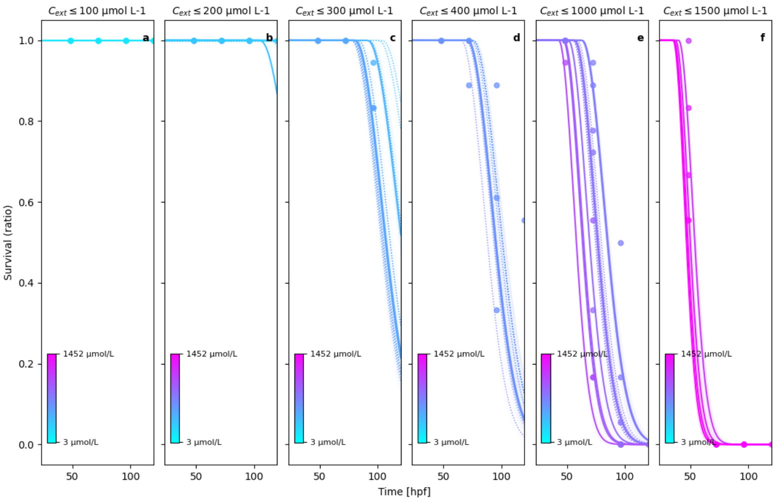

Figure S19. Posterior estimates and 95%-BCIs of naproxen for GUTS-reduced damage model.

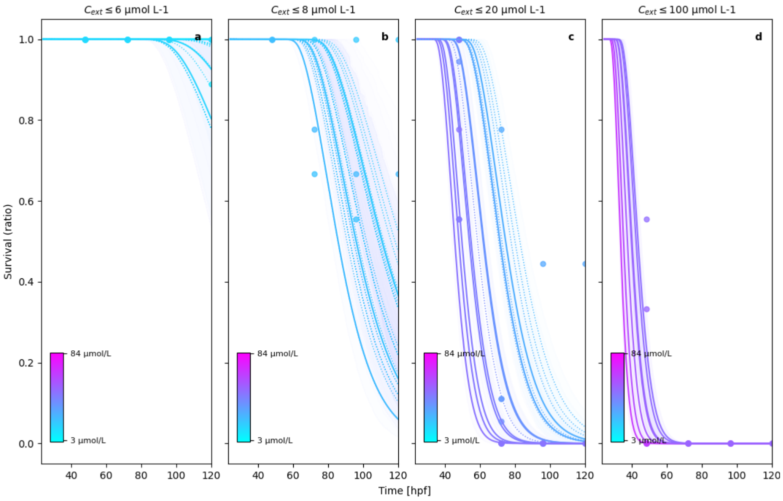

Figure S20. Posterior estimates and 95%-BCIs of diclofenac for GUTS-reduced damage model.

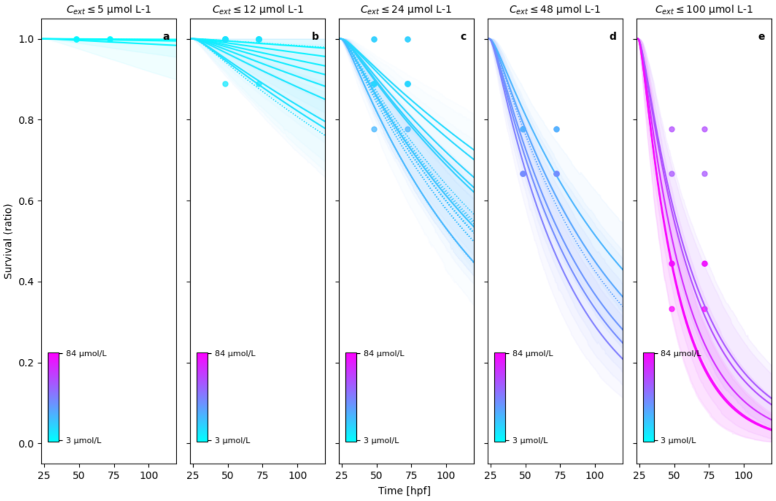

Figure S21. Posterior estimates and 95%-BCIs of diuron for GUTS-reduced damage model.

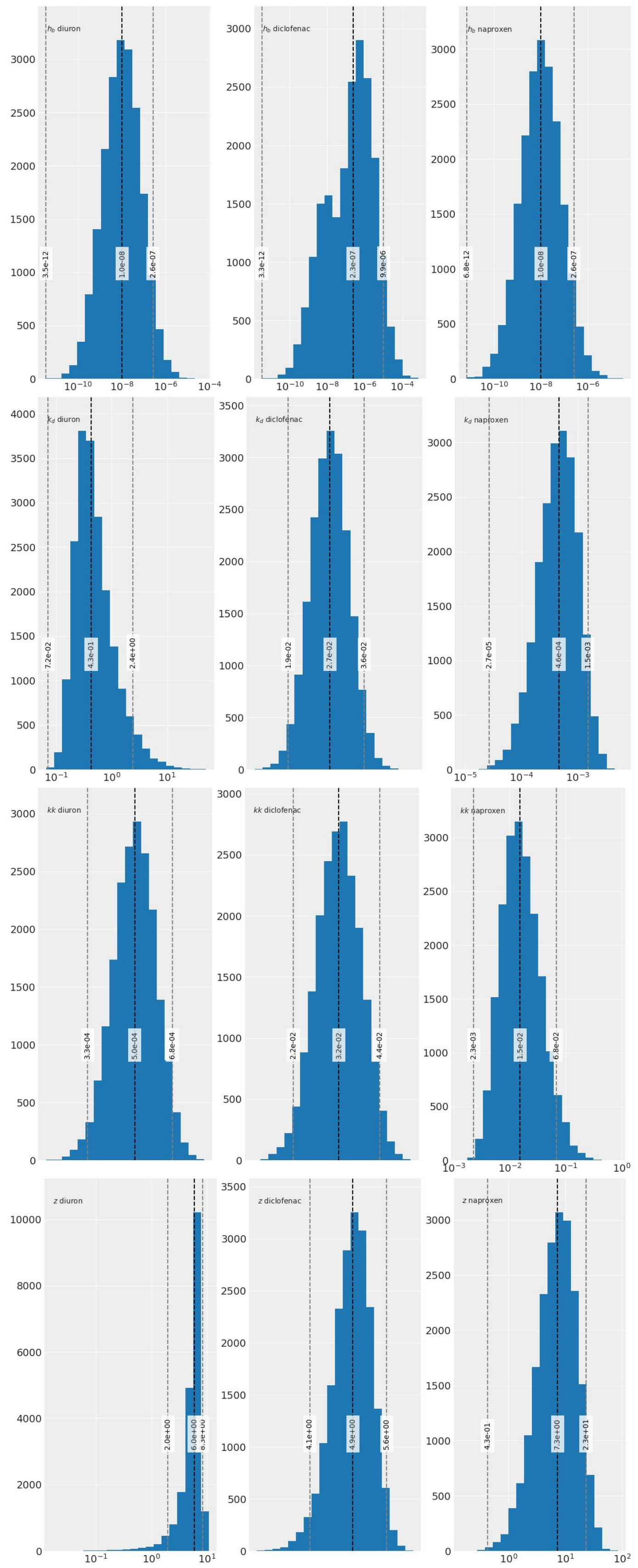

Figure S22. Parameter estimates of the GUTS-reduced model with substance specific parameters.

Table S10. Parameter estimates and posterior highest densitiy intervals (HDI) of the GUTS-reduced model. The HDI contains 94% of the probable parameter values given the data.

Parameters		Diuron			Diclofenac			Naproxen	
	mean	hdi 3%	hdi 97%	mean	hdi 3%	hdi 97%	mean	hdi 3%	hdi 97%
k_d (h^{-1})	0.80	0.08	2.17	0.03	0.02	0.04	0.00	0.00	0.00
z $(\mu mol\ L^{-1})$	5.60	2.20	8.35	4.85	4.11	5.59	9.24	0.43	22.17
k_k $(\frac{L}{\mu mol\ h})$	0.00	0.00	0.00	0.03	0.02	0.04	0.02	0.00	0.06
h_b (h^{-1})	0.00	0.00	0.00	0.00	0.00	0.00	0.00	0.00	0.00

S12 Estimated half-life of nrf2

The half-life of *nrf2* has been estimated at approximately 20 minutes [11]. This value was compared against the posterior parameter distribution of the RNA-decay rate constant k_{rd}, inserting it in a simple exponential decay equation $R(t) = R_0^{-k_{rd}\ t}$ and solving for t at which $R(t) = R_0/2$, which gives the half-life of $t_{1/2} = \frac{ln(2)}{k_{rd}}$.

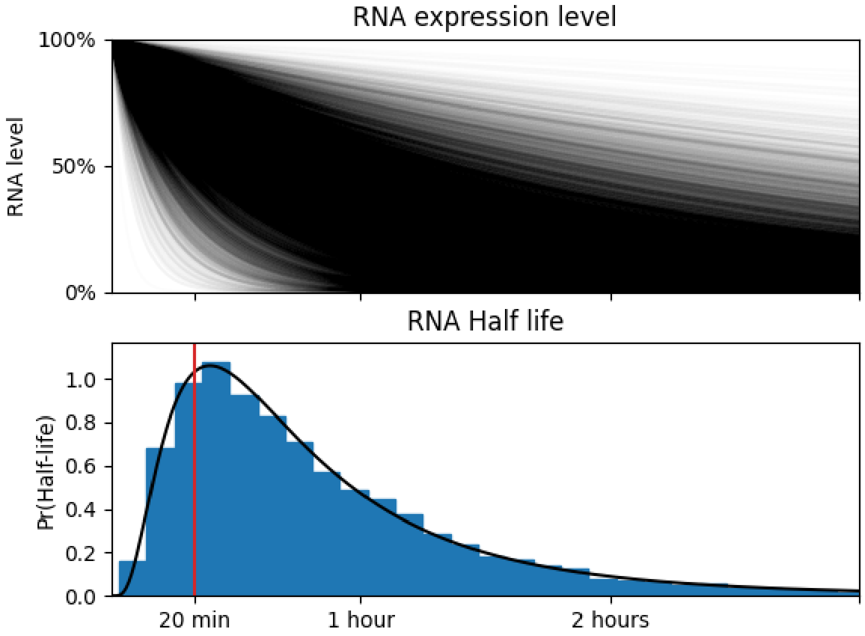

Figure S23. Estimated half-life of RNA expression from the r_{rd} parameter. A simple exponential decay model was assumed to estimate the half life. And a log-normal probability distribution was fitted to estimate the distribution of half-life times.

S13 Estimated half-life of proteins

The half-life of proteins is estimated to lie between 20–46 hours [12]. This value was compared against the posterior parameter distribution of the dominant rate constant for protein dynamics k_p, inserting it in a simple exponential decay equation $P(t) = P_0^{-k_p\ t}$ and solving for t at which $P(t) = P_0/2$, which gives the half-life of $t_{1/2} = \frac{ln(2)}{k_p}$. Although the dominant rate constant lumps protein synthesis and decay together into one constant, it can give an idea of the approximate timescale of the dynamics. Seeing that the estimated half-life distribution matches the literature data to some degree. Can be seen as a confirmation that the approximate dynamics is captured correctly. Nevertheless, the true kinetics can only be estimated, once protein measurements are integrated into the model.

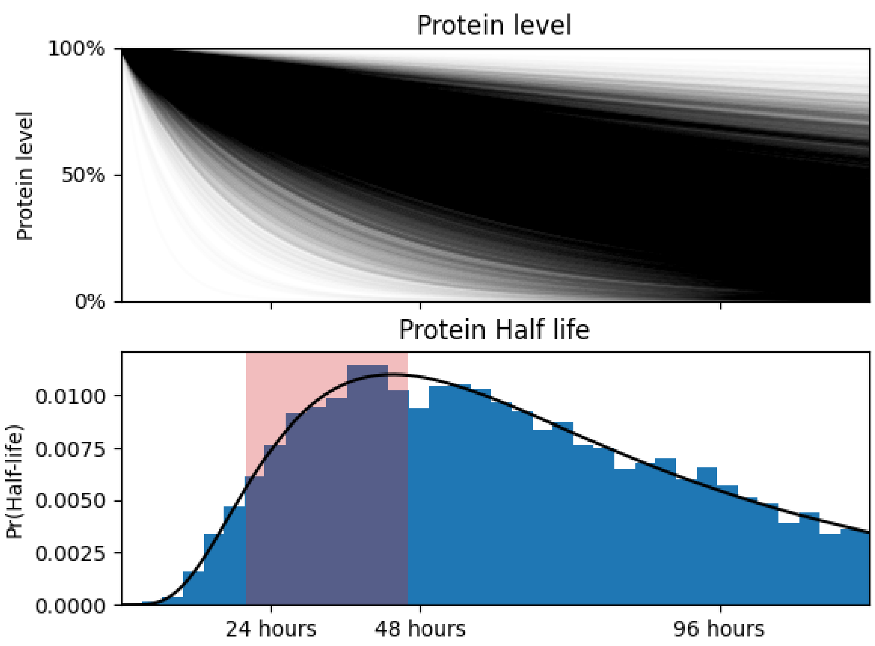

Figure S24. Estimated half-life of proteins from the r_p parameter. A simple exponential decay model was assumed to estimate the half-life. And a log-normal probability distribution was fitted to estimate the distribution of half-life times.

References

[1] Nir Yosef and Aviv Regev. "Impulse Control: Temporal Dynamics in Gene Transcription". In: *Cell* 144.6 (Mar. 2011), pp. 886–896. ISSN: 00928674. DOI: 10.1016/j.cell.2011.02.015. URL: https://linkinghub.elsevier.com/retrieve/pii/S0092867411001292 (visited on 10/13/2023).

[2] Ziv Bar-Joseph, Anthony Gitter, and Itamar Simon. "Studying and Modelling Dynamic Biological Processes Using Time-Series Gene Expression Data". In: *Nature reviews. Genetics* 13.8 (Jan. 1, 2012), pp. 552–64. DOI: 10.1038/nrg3244.

[3] Tjalling Jager et al. "General Unified Threshold Model of Survival–a Toxicokinetic-Toxicodynamic Framework for Ecotoxicology". In: *Environmental science & technology* 45.7 (Jan. 1, 2011), pp. 2529–40. DOI: 10.1021/es103092a.

[4] Tjalling Jager and Roman Ashauer. *Modelling Survival under Chemical Stress: A Comprehensive Guide to the GUTS Framework*. 2nd ed. Leanpub, Jan. 2018. ISBN: 978-1-9999705-1-2.

[5] Richard McElreath. *Statistical Rethinking: A Bayesian Course with Examples in R and Stan*. 1st ed. New York: Chapman and Hall/CRC, 2016. ISBN: 978-1-315-37249-5.

[6] Eli Bingham et al. *Pyro: Deep Universal Probabilistic Programming*. Oct. 18, 2018. URL: http://arxiv.org/abs/1810.09538 (visited on 03/26/2024).

[7] Du Phan, Neeraj Pradhan, and Martin Jankowiak. "Composable Effects for Flexible and Accelerated Probabilistic Programming in NumPyro". In: *arXiv* (Dec. 24, 2019). URL: http://arxiv.org/abs/1912.11554 (visited on 03/26/2024).

[8] James Bradbury et al. *JAX: Composable Transformation of Python+NumPy Programs*. Version 0.3.13. 2018. URL: http://github.com/google/jax.

[9] Matthew D. Hoffman and Andrew Gelman. "The No-U-Turn Sampler: Adaptively Setting Path Lengths in Hamiltonian Monte Carlo". In: *arXiv* (Nov. 17, 2011). URL: http://arxiv.org/abs/1111.4246 (visited on 12/14/2023).

[10] David M. Blei, Alp Kucukelbir, and Jon D. McAuliffe. "Variational Inference: A Review for Statisticians". In: *Journal of the American Statistical Association* 112.518 (Apr. 3, 2017), pp. 859–877. ISSN: 0162-1459, 1537-274X. DOI: 10.1080/01621459.2017.1285773. URL: https://www.tandfonline.com/doi/full/10.1080/01621459.2017.1285773 (visited on 04/02/2024).

[11] Akira Kobayashi et al. "Oxidative Stress Sensor Keap1 Functions as an Adaptor for Cul3-Based E3 Ligase To Regulate Proteasomal Degradation of Nrf2". In: *Molecular and Cellular Biology* 24.16 (Aug. 1, 2004), pp. 7130–7139. ISSN: 1098-5549. DOI: 10.1128/MCB.24.16.7130-7139.2004. URL: https://www.tandfonline.com/doi/full/10.1128/MCB.24.16.7130-7139.2004 (visited on 11/16/2023).

[12] J. Wade Harper and Eric J. Bennett. "Proteome Complexity and the Forces That Drive Proteome Imbalance". In: *Nature* 537.7620 (Sept. 2016), pp. 328–338. ISSN: 0028-0836, 1476-4687. DOI: 10.1038/nature19947. (Visited on 03/12/2024).
